# Genome-Wide Prediction, Functional Divergence, and Characterization of Stress-Responsive *BZR* Transcription Factors in *B. napus*

**DOI:** 10.3389/fpls.2021.790655

**Published:** 2022-01-04

**Authors:** Rehman Sarwar, Rui Geng, Lei Li, Yue Shan, Ke-Ming Zhu, Jin Wang, Xiao-Li Tan

**Affiliations:** ^1^School of Food Science and Biological Engineering, Jiangsu University, Zhenjiang, China; ^2^School of Life Sciences, Jiangsu University, Zhenjiang, China

**Keywords:** *Brassica napus*, brassinosteroid, miRNAs, abiotic stress, gene expression

## Abstract

BRASSINAZOLE RESISTANT (BZR) are transcriptional factors that bind to the DNA of targeted genes to regulate several plant growth and physiological processes in response to abiotic and biotic stresses. However, information on such genes in *Brassica napus* is minimal. Furthermore, the new reference *Brassica napus* genome offers an excellent opportunity to systematically characterize this gene family in *B. napus*. In our study, 21 *BnaBZR* genes were distributed across 19 chromosomes of *B. napus* and clustered into four subgroups based on *Arabidopsis thaliana* orthologs. Functional divergence analysis among these groups evident the shifting of evolutionary rate after the duplication events. In terms of structural analysis, the *BnaBZR* genes within each subgroup are highly conserved but are distinctive within groups. Organ-specific expression analyses of *BnaBZR* genes using RNA-seq data and quantitative real-time polymerase chain reaction (qRT-PCR) revealed complex expression patterns in plant tissues during stress conditions. In which genes belonging to subgroups III and IV were identified to play central roles in plant tolerance to salt, drought, and *Sclerotinia sclerotiorum* stress. The insights from this study enrich our understanding of the *B. napus BZR* gene family and lay a foundation for future research in improving rape seed environmental adaptability.

## Introduction

Abiotic and biotic stresses are the two main environmental factors that influence plant development and quality throughout their life span. Transcriptional factors (TFs) play an indispensable role in mediating multiple hormonal signaling pathways to drive adaptive response against abiotic and biotic stresses. It is well known that a group of transcriptional factors, BRASSINAZOLE RESISTANT (BZR), bind to specific sequences to convey environmental stimuli in regulating plant tolerance to various stresses (Glazebrook, 2001). BZR, also named BRI1-EMS-SUPPRESSOR (BES), is a positive regulator of the brassinosteroid (BR) hormone. BR is the polyhydroxy steroid hormone required in various plant development processes, including photomorphogenesis, cell division, vascular differentiation, pollen tube formation, seed germination, reproduction, elongation, senescence, and exhibits response to various abiotic stresses (Zhu et al., 2013; Fridman et al., 2014; Wei and Li, 2016; Li et al., 2018). It is also noteworthy that the exogenous application of BR can act as an immunomodulator to improve plant tolerance against biotic stresses, including bacterial and pathogen infection (He et al., 2007; Chinchilla et al., 2009). BR mediates plant development through BRASSINOSTEROID-INSENTIVE 1 (BRI1), a membrane-localized receptor, which upon binding with BRI1-ASSOCIATED RECEPTOR KINASE (BAK1), directs the transcriptional activity of the BZR TFs (Li et al., 2001; He et al., 2002; Yin et al., 2002; Kim et al., 2011). BZR transcription family contains two homologous TFs, BRASSINAZOLE RESISTANT 1 (BZR1), and BRASSINOSTEROID INSENSTIVE 1-ETHYLE METHAESULFONATE SUPPRESSOR 1 (BES1/BZR2), which are known to be the central transcription factors in BR signaling. BZR TFs are mainly expressed in leaf, flower, root, and shoot (Li and Chory, 1999; Reise and Waller, 2009) and are localized in the nucleus to bind with the E-box (CANNTG) and BR-response (CGTGT/CG) *cis*-elements of one-quarter of BR-responsive genes (Yu et al., 2011; Zhang et al., 2014; Wu et al., 2016b). These interactions coordinate various aspects of molecular and cellular processes, including plant tolerance to abiotic stresses. For instance, BZR1 interacts with C-REPEAT/DRE BINDING FACTOR (CBF) to govern freezing tolerance in *Arabidopsis thaliana* (Li et al., 2017). At the same time, *BZR2* directly binds with the promoter region of glutathione s-transferase-1 (*GST1*) to maintain plant growth under drought stress (Cui et al., 2019). Moreover, BZR1 also binds with FLOWERING LOCUS D (FLD) to regulate flowering (Zhang et al., 2013b). Furthermore, recent studies have also reported the interaction of BZR1/BZR2 with PHYTOCHROME INTERACTING FACTORS (PIFs), MAP KINASE 6 (MAPK6), WRKY TRANSCRIPTION FACTOR 54 (WRKY54), ATBS1-INTERACTING FACTOR 2 (AIF2), and transcription elongation factor IWS1 to mediate BR-related plant development and defense-related processes (Li et al., 2010; Oh et al., 2012; Kang et al., 2015; Chen et al., 2017; Kim et al., 2017; Jia et al., 2020). Subsequent studies indicate that BZR TFs also show higher affinity with transcriptional factors of multiple phytohormones. For instance, *BZR1* and *BZR2* interact with BRI1-like receptor gene 3 (*BRL3*) to suppress the transcriptional activity of the positive regulator of abscisic acid (ABA) to inhibit seed dormancy and increase flowering growth during the reproductive stage (Ryu et al., 2014; Yang et al., 2016). Additionally, *BZR1* and *BZR2* also bind with the negative regulator of gibberellins signaling to arbitrate plant growth in response to relentless stresses (Li et al., 2012). These molecular interactions between transcriptional factors of the different phytohormones evidenced the crosstalk between BR and multiple phytohormone signaling. However, the precise molecular mechanism of these synergies is mainly unknown.

In *Arabidopsis*, six members of the *BZR* gene family were identified, *AtBZR1, AtBZR2, AtBEH1, AtBEH2, AtBEH3,* and *AtBEH4*. The dominant mutants of *bzr1-D* and *bes1-D* displayed a delay in flowering with wider dark green leaves and showed upregulated expression of BR-responsive genes in *A. thaliana* (Wang et al., 2002; Yin et al., 2002). Additionally, a recent study also confirmed the function of *BZR* in light signaling pathways, which shows the interaction of *BZR1* with light-regulated transcriptional factor HY5 in regulating plant photomorphogenesis (Li and He, 2016), whereas downregulation of wheat *BZR2* displays sensitivity to drought stress (Cui et al., 2019). In contrast, *BEH1-4* genes are thought to have a partially redundant function in BR signaling. For instance, in *A. thaliana*, the quadruple mutant (*beh1, beh2, beh3*, and *beh4*) did not exhibit any noticeable phenotype, while sextuple mutant (*bzr1, bes1, beh1, beh2, beh3*, and *beh4*) shows abnormal tapetum development, and male sterility (Chen et al., 2019a,b). However, a recent study listed the function of *AtBEH4* in modulating embryonic stem development (Lachowiec et al., 2018), suggesting the redundant functions of the *BZR* gene family. However, there is insufficient progress in understanding the individual role of *BEH1-4* genes, especially in abiotic and biotic stresses. Overall, significant progress has been made to systematically characterize the *BZR* gene family in *Oryza sativa* (Tong et al., 2012), *Zea mays* (Manoli et al., 2018), *Brassica rapa* (Saha et al., 2015), *Glycine max* (Zhang et al., 2016), *Solanum lycopersicum* (Liu et al., 2014), *Petunia hybrida* (Verhoef et al., 2013), *Eucalyptus grandis* (Fan et al., 2018), and other plant species (Table 1), which shows various functions of the *BZR* genes in regulating important agronomic traits including tillering, stress tolerance, and yield. However, no progress has been made to characterize the functional divergence of the *BZR* gene family and their regulatory mechanism in *Brassica napus*.

**Table 1:** Number of *BZR* genes in *Brassica napus* and other plant species.

Species name	Common name	Genome size Mb	Total number of chromosomes	Number of *BZR* genes	References
*Brassica napus*	Rape seed	1,132	38	21	This study
*Arabidopsis thaliana*	Thale cress	135	5	6	He et al., 2005; Yu et al., 2011
*Solanum lycopersicum*	Tomato	950	12	9	Liu et al., 2014; Su et al., 2021; Wang et al., 2021
*Brassica rapa*	Bird rape	485	16	15	Saha et al., 2015; Wu et al., 2016a
*Glycine max*	Soybean	1,150	10	4	Zhang et al., 2016
*Gossypium hirsutum*	Cotton	2,500	26	14	Guo et al., 2017
*Eucalyptus grandis*	Rose gum	640	11	6	Fan et al., 2018
*Zea mays*	Maize	2,135	10	11	Manoli et al., 2018; Yu et al., 2018
*Beta vulgaris* L	Sugar beat	758	18	6	Wang et al., 2019
*Malus domestica*	Apple	750	17	22	Cao et al., 2020; Jiang et al., 2021
*Nicotiana benthamiana*	Tobacco	3,000	19	14	Chen et al., 2021
*Triticum aestivum* L	Wheat	17,000	42	20	Kesawat et al., 2021; Liu et al., 2021
*Ammopiptanthus nanus*	Relict-xerophyte shrub	88,892	9	8	Ding et al., 2021

*Brassica napus* is one of the few dicot plants that have very valuable uses. Its oil-rich seed is utilized for industrial and nutritional purposes or as a protein-rich forage for animals (Friedt et al., 2018). However, *B. napus* growth is sensitive to a variety of biotic and abiotic stresses, including waterlogging, salt, drought, and *Sclerotinia sclerotiorum* infection (Xu et al., 2015; Sun et al., 2017; Hatzig et al., 2018; Sabagh et al., 2019), which significantly affects *B. napus* economic interest. Furthermore, its complex polyploid genome makes it challenging to understand the genetic variations underlying *B. napus* resistance to abiotic and biotic stresses.

This study comprehensively described the *B. napus BZR* gene family based upon their evolutionary relationship, structural features, and protein–protein interactions. Moreover, miRNA targets and promoter analyses were also performed. Additionally, expression patterns in response to salt and drought stress were also examined by qRT-PCR. The data present in our study showed that 21 *BnaBZRs* were scattered throughout 19 chromosomes of *B. napus* and organized into four subgroups. Functional divergence analysis among these groups evident the shifting of evolutionary rate after the duplication events. Organ-specific expression analysis of *BnaBZR* genes using RNA-seq data and qRT-PCR showed complex expression patterns in plant tissues (roots, mature siliqua, leaf, flower, flower-bud, stem, and seed) and during stress conditions. In which genes belonging to subgroups III and IV were identified to play central roles in plant tolerance to salt, drought, and *S. sclerotiorum* stress. In addition, we also explore some bna-miRNA, such as bna-miRNA395, bna-miR171, and bna-miR159, which are stimulated by the environmental cues, suggesting that different bna-miRNAs direct post-transcriptional regulation of *BnaBZR* gene family during biotic and abiotic stresses. Insights from these findings provide a framework for future studies in improving rape seed environmental adaptability.

## Materials and Methods

### Prediction of *BZR* Genes in *Brassica napus*

The proteins sequences of the *B. napus* BZR genes were downloaded from the brassica genome browser GENOSCOPE^1^ (Chalhoub et al., 2014) and BnPIR^2^ (Song et al., 2020, 2021), respectively, by employing the six *AtBZR* genes from *A. thaliana* genome browser TAIR^3^ with corresponding Gene ID (*At1G19350, At1G75080, At3G50750, At4G36780, At4G18890*, and *At1G78700*). Furthermore, the amino acid sequences from *B. oleracea* and *B. rapa* were acquired from the *Brassica* database BRAD,^4^ using the *AtBZRs* as a query. The protein sequence with 80% sequence similarity was uploaded to ScanProsite^5^ (De Castro et al., 2006) and Pfam^6^ (Mistry et al., 2021) to search for the presence of the BZR1 domain in the sequence, protein sequences with lacking distinctive matching domains were eliminated. Moreover, multiple sequence alignment analysis was performed by aligning the 46 BZRs protein sequence from *A. thaliana*, *B. oleracea*, *B. napus*, and *B. rapa* to confirm the conserved domain. Physiochemical characteristics of the BnaBZRs proteins were investigated by the ProtParm^7^ tool (Gasteiger et al., 2005) and the cellular location was predicted by Plant-mPLoc^8^ (Chou and Shen, 2010).

### Phylogenetic, Gene Structure, and Motif Distribution Analyses of the *BnaBZRs*

The NJ (Neighbor-joining) method in MEGA X was utilized to plot the evolutionary tree. The tree’s validity was verified by performing 1,000 boot replications, and the Newick format of the phylogenetic tree was further annotated and visualized by the iTOL^9^ web server (Letunic and Bork, 2021). The genes structural features were recognized by uploading the coding and genomic sequences in the gene structure display server GSDS^10^ (Hu et al., 2015) and FGENESH (Solovyev et al., 2006). To further verify the intron/exon arrangement, we manually aligned the genomic and coding sequences of each *BZR* gene. Functional conserved motifs were examined by using the MEME^11^ program (Bailey and Elkan, 1994) with the minimum motif search configured to 20, and the rest of the settings left to default.

### Chromosomal Distribution, Gene Duplication, and Site-Specific Assessment of *BZR* Gene Family in *Brassica napus*

The genome feature file (GFF) of the *B. napus* genome was employed to inspect the chromosomal distribution of the 21 *BnaBZRs*, and the map was drawn using the TBtools program (Chen et al., 2020). Moreover, Gene duplication events in *BnaBZR* genes were performed using the MCScanX toolkit (Wang et al., 2012b). The collinear relationship between *BnaBZR* genes and *BZR* genes from *B. oleracea*, *A. thaliana*, and *B. rapa* were acquired by Dual Synteny Plotter function in TBtools. Moreover, positive and purifying selection of the *BZR* gene family was predicted by utilizing a Selecton Server^12^ (Stern et al., 2007). Besides this, the nonsynonymous (*Ka*) and synonymous mutation (*Ks*) of a single codon were also calculated by *Ka/Ks* Calculator 2.0 (Wang et al., 2010).

### Functional Divergence and 3D Structure Prediction of BZR in *Brassica napus*

To determine the Type-I and Type-II functional divergence among clusters of the *BnaBZRs*, we used the DIVERGE 3.0 program (Gu et al., 2013), and Bayesian posterior probability *Q_k_* was set to 0.9 to predict the specific amino acid site. Protein 3D structure was generated by submitting the *BnaA06BZR1* protein sequence into the I-TASSER server^13^ with default parameters (Yang et al., 2015), and specific amino acid sites were marked and visualized using the PyMOL software.^14^

### Plant Material, Stress Treatment, RNA Isolation, and qRT-PCR Analyses

Seeds of *B. napus* cultivar Zhongshuang 11 (ZS11) were obtained from the Oil Crops Research Institute of the Chinese Academy of Agricultural Sciences, Wuhan. The seeds were sprouted on moistened filter paper under the listed conditions: 70% relative humidity and 20 ± 5°C, 16 h light/8 h dark at a light intensity of 50 μmol/m^2^/s. To investigate the expression profile of *BnaBZRs* in *B. napus*, tissues from the adult plant, such as mature-siliqua, root, stem, seed, leaf, flower, flower-bud, and shoot-apex, were collected and stored at −80°C for further experiment. Furthermore, for salt treatment, the three-week-old seedlings were immersed into 400 mM sodium chloride (NaCl; received 100 mM NaCl per day). After reaching a final concentration (400 mM), the leaf samples were collected at 2, 8, and 16 h post-treatment, while for drought treatment, 25% PEG solution was applied to simulate a drought stress-like condition. The leaf tissues were harvested at 2, 8, and 16 h post-treatment. The collected leaves were instantly frozen into the LN2 (liquid nitrogen) and kept at −80°C. To extract RNA and synthesize cDNA, we employed the same procedure as described in our prior study (Sarwar et al., 2021). The synthesized cDNA of the individual sample was utilized as a template in the Thermo Fisher QuantStudio Real-Time PCR system with three separate biological replicates for qRT-PCR analysis. For internal control, the *B. napus Actin* gene (geneBank ID: XM013858992) was used. The expression patterns of *BnaBZR*s were measured by the 2^−∆∆Ct^ method described by Livak and Schmittgen (2001), and a student *t-*test was performed to find the significant difference. The graph was generated using GraphPad Prism8.0 software (Swift, 1997). The qRT-PCR primers were designed according to the qPCR SYBR-Green Master Mix instruction and listed in Supplementary Table S12.

### Protein–Protein Interaction, miRNA Prediction, *cis*-Element Regulatory, and Gene Ontology Analyses

For protein–protein interaction analysis, the *AtBZRs* orthologs were searched in the STRING server^15^ (Szklarczyk et al., 2019), and the interaction map was drawn using the Cytoscape program. Moreover, to confirm the interaction of miRNA with *BnaBZRs*, we used the publicly available *B. napus* miRNAs from miRbase^16^ (Kozomara et al., 2019) and PNRD^17^ (Yi et al., 2015), and searched against the identified 21 coding sequences of *BnaBZRs* in psRNAtarget server^18^ (Dai and Zhao, 2011). Furthermore*, cis*-elements were investigated by using the 1.5 bp genomic promoter sequence of the individual *BnaBZR* gene and uploaded to the plantCARE database^19^ (Lescot et al., 2002). Additionally, the functional properties of the *BnaBZRs* were evaluated using the *AtBZRs* orthologs in DAVID (Huang et al., 2009a,b) and PANTHER (Mi et al., 2019) program, GO terms with significant value *p* < 0.05 were considered to be enriched in *BZR* genes.

### Expression Pattern and SNPs Distribution Analysis of the *BnaBZRs*

The expression data of *BnaBZRs* under drought, salinity, cold, and *S. sclerotiorum* stress treatment were acquired from the publically available RNA-seq data sets (CRA001775; Lin et al., 2020) and (SRP311601; Walker et al., 2021). DSEeq2 package in R-studio was applied to measure the genes differential expression, the detected values were adjusted by log2 fold change method, and heatmap graph was created by the TBtools program. To locate the sequence polymorphism of the *B. napus BZR* gene family, the SNPs from the *BnaBZRs* of 159 accessions were extracted from the *B. napus* genomic browser BnPIR^2^ (Song et al., 2020, 2021), and the SNP distribution map was drawn using the TBtools.

## Results

### Detection and Annotation of *Brassica napus BZR* Genes

The *BZR* gene family in *A. thaliana* and other plant species has been studied extensively. However, there are no studies available that have characterized the *B. napus BZR* genes in depth. Therefore, to examine the *B. napus BZR* genes, we obtained the peptide sequences of the *AtBZR* gene family (*AtBZR1, AtBZR2, AtBEH1, AtBEH2, AtBEH3,* and *AtBEH4*) from the *A. thaliana* genome browser and then used it as a query in BLASTP program in the *B. napus* genome browser GENOSCOPE (see footnote 1; Chalhoub et al., 2014). Moreover, the BnPIR (see footnote 2) *B. napus* genomic browser was also utilized to further verify the reliability of the *BZR* gene family, and redundant sequences were eliminated manually. A total of 21 *BnaBZRs* were recognized, including seven *BnaBZRs* and 14 *BnaBEH*s, which were named according to their loci (Supplementary Table S1). Simultaneously, seven and 12 *BZR*s were recognized in the genome of *B. oleracea* and *B. rapa*, respectively. This exhibits that the *BnaBZR* gene family might derive from their ancestor *B. rapa* and *B. oleracea*. Moreover, physicochemical properties of the BnaBZR proteins reveal that the instability index (II) was among 46.64–85.28, which shows that members of BnaBZRs were unstable. In addition, the isoelectric point (pI) values were between 5.14 and 9.81, indicating BnaBZRs were highly basic, and the negative GRAVY (grand average of hydrophobicity) value shows that these proteins are hydrophilic in nature. The amino acids encoded by the BnaBZR proteins are varied from 102aa to 400aa, in which the BnaA01BEH3 amino acid sequence was the shortest contained only 102 amino acids; however, the amino acid length of BnaC08BZR2-1 was the highest, around 400 amino acid residues. The molecular weight ranges from 10,990 KD to 44,156 KD, and the aliphatic index (AI) was between 48.12 and 73.3. Moreover, the subcellular localization signals of BnaBZRs were predicted by Plant-mPLoc. As transcriptional regulatory proteins, all BnaBZRs signals were detected in the nucleus (Supplementary Table S1).

### Phylogenetic and Structural Prediction of *BnaBZRs*

**Figure 1:**
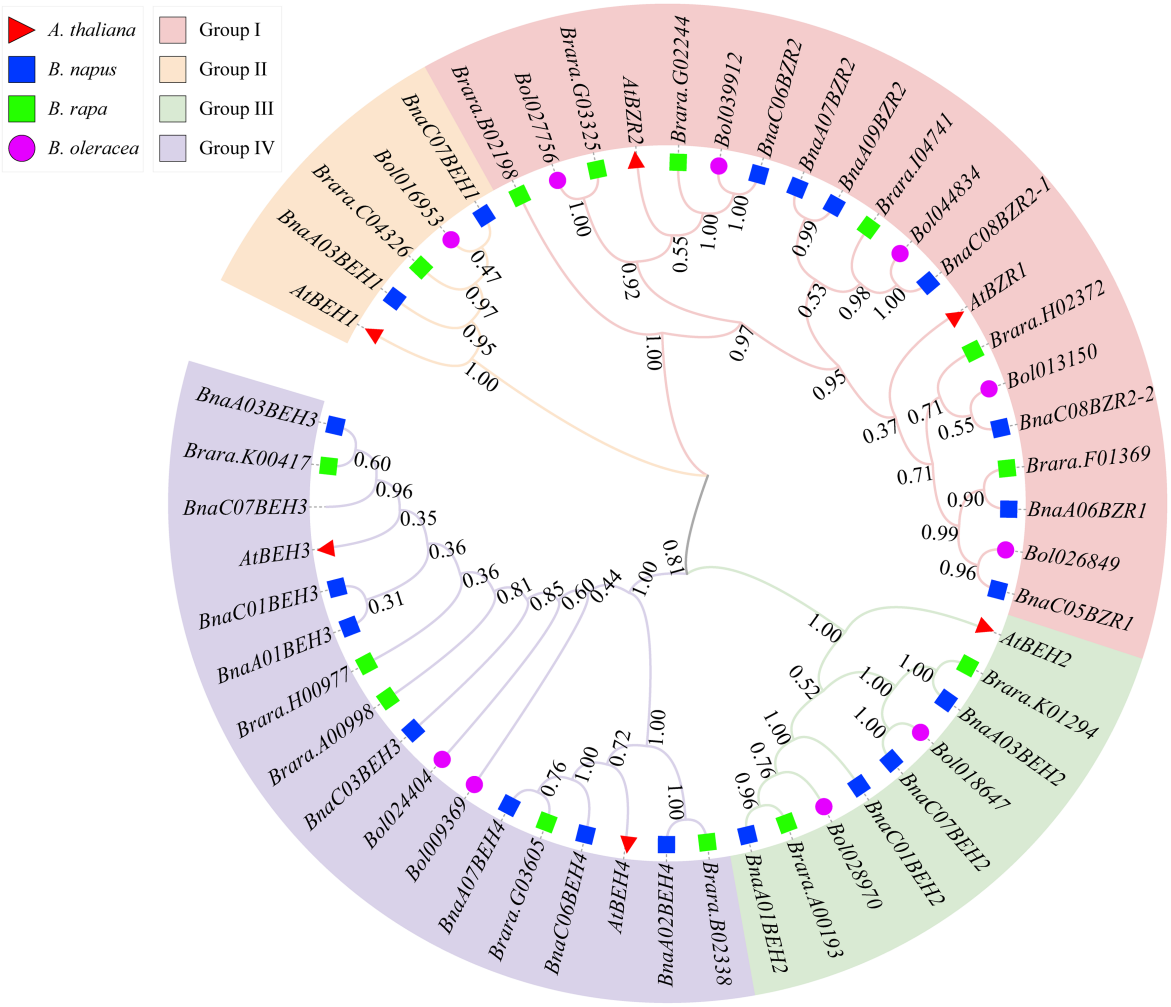
Phylogenetic relationship of the *BZR* gene family from *Brassica napus* (*Bna*), *Arabidopsis thaliana* (*At*), *B. rapa* (*Bra*), and *B. oleracea* (*Bol*). Neighbor-joining (NJ) tree was constructed using the MEGA X with 1,000 bootstrap replication and visualized using online tool iTOL. Members in the *BZR* gene family clustered into the four groups and specified with different colors.

**Figure 2:**
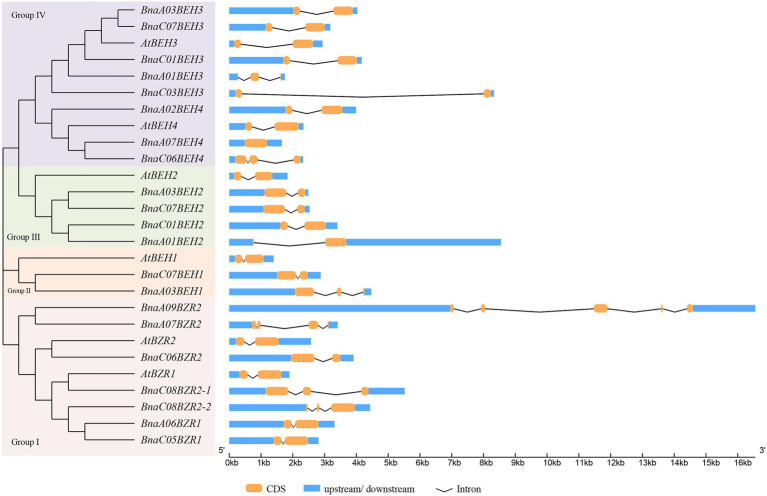
Gene structure analysis of the *B. napus* and *A. thaliana BZR* gene family. Each *BZR* gene subfamily indicated as different color in *Y*-axis, while the *X*-axis represented the lengths of the exons and introns.

To explain the phylogenetic relationship of the *BnaBZRs*, an evolutionary tree was generated by aligning the full-length coding sequence of six *A. thaliana*, 21 *B. napus*, 12 *B. rapa*, and seven *B. oleracea BZR* genes. As shown in Figure 1, all 47 genes were fell into four groups: group I carried *BZR1* and *BZR2* genes; group II contained *BEH1*; and group III contained *BEH2* genes, while group IV contained *BEH3* and *BEH4* genes. Interestingly, the homologs of *BEH3* and *BEH4* were not detected in the *B. oleracea* related to those of *A. thaliana*, *B. rapa*, and *B. napus* indicating that the *BZR* gene family in *B. oleracea* might experience an evolutionary divergence in structural features. Additionally, the expansion of the *BEH* genes in *A. thaliana*, *B. napus*, and *B. rapa* take place in group II, III, and group IV due to whole-genome duplication. We also found that each *BnaBZR* gene shows higher homology with *A. thaliana BZR* genes. For instance, group I carried *AtBZR1* and *AtBZR2*, along with *BnaA06BZR1, BnaC05BZR1,* and *BnaC08BZR2-1, BnaC08BZR2-2, BnaC06BZR2*, whereas *BnaBEH* genes were highly conserved with the *A. thaliana BEH* genes. Overall, in *B. napus*, group I, II, III, and IV had seven, two, four, and eight *BZR* family members, respectively. The genes cluster within the same subfamily displays a similar role in *B. napus*. Furthermore, to understand the diversity of *BnaBZRs*, Gene Structure Display (GSDS) analysis was performed using the coding and genomic sequence of the *B. napus* and *A. thaliana*. As shown in Figure 2, one to five exons were predicted in the *BnaBZRs.* Similar to the *A. thaliana BZR* gene family, most members in the *BnaBZRs* subfamily had the same number of exons and intron. For instance, most members of group I, group II, and group III have two to three exons and one to two introns, except for *BnaA09BEH2*, which contains four introns. Conversely, group IV contained one to three exons and only one intron, except for *BnaA07BEH4*, with no intron. Results from this analysis indicate that group I, II, and III might receive the original genes because, in the previous studies, it has been reported that the gain of introns is far slower than the intron loss during duplication (Nuruzzaman et al., 2010). Therefore, members in group IV might be derived from the original genes during evolutionary processes.

### Functional Divergence Analysis of *BnaBZR* Gene Family

Our study observed that the *B. napus BZR* gene family was grouped into four groups. To determine the potential functional divergence between groups, we tested the Type I (*ϴ*I) and Type II (*ϴ*II) functional divergence utilizing the CodeML package in DIVERGE v2.0 (Gu and Vander Velden, 2002). For this analysis, we use group I, III, and IV members, while group II was excluded because it comprises only two members of the *BnaBZRs*. The results indicate that the all-divergence coefficients of Type I among group I/III, group I/IV, and group III/IV were statistically significant, with the *ϴ*I and LRT values varying from 0.99 to 0.646 and 0.86 to 33.9, respectively, indicating that the amino acid sites among different groups of *BnaBZR* gene family have altered evolutionary constraints, which lead to group-specific functional evolution after divergence (Table 2). In contrast, the coefficients of Type II among group I/IV and group III/IV were also statistically significant with an *ϴ*II value of 0.319 and 0.082, respectively, indicating the radical shift in amino acids properties of these pairs during evolution. However, the coefficient of *ϴ*II among group I/III was not significant. We also found some crucial amino acid residues sites that may affect the functional divergence of the *B. napus BZR* gene family. To reduce false-positive results, posterior probability (*Q_k_* > 0.9) was selected to detect specific amino acid residues among the *BnaBZR* family (Table 2). The results predict 21 similar Type-I sites among group I/III and group I/IV. In comparison, only two sites were examined in group III/IV, implying that significant functional divergence might exist in group I/III and group I/IV as compared to group III/IV. Conversely, no Type-II functional divergence sites were predicted in group I/III and group I/IV. However, three sites occurred in group III/IV, of which 99T, 100R, and 155S were examined in both *ϴ*I and *ϴ*II, indicating the altered selective constraints may happen at these sites.

**Table 2:** Functional divergence analysis between different groups of the *BnaBZR* gene family.

Cluster 1	Cluster 2	Type I				Type II		
		*ϴ*I ± SE	LRT	*Q_k_* > 0.8	Critical amino acids	*ϴ*II ± SE	*Q_k_* > 0.8	
Group I	Group III	0.99 ± 1.075	0.862	21	**19T, 20R,** 21R, 22K, 23P, 24S, 25W, 59K, 60H, 72S, 73E, 85Y, 86R, 321L, 322E, 323L, 324T, 325L, 308W, 309E, 310G	−0.259 ± 0.344	0	None
Group I	Group IV	0.99 ± 0.171	33.9	21	**19T, 20R**, 21R, 22K, 23P, 24S, 25W, 59K, 60H, 72S, 73E, 85Y, 86R, 321L, 322E, 323L, 324T, 325L, 308W, 309E, 310G	0.319 ± 0.186	0	None
Group III	Group IV	0.646 ± 0.224	8.316	2	**19T, 20R**	0.085 ± 0.102	3	**155S, 19T, 20R**

LRT, likelihood ratio statistics; *Q_k_*, posterior probability; and Sites in the bold are responsible for type-I and type-II functional divergence.

### 3D Structure and Critical Amino Acid Site Prediction of BZR in *Brassica napus*

**Figure 3:**
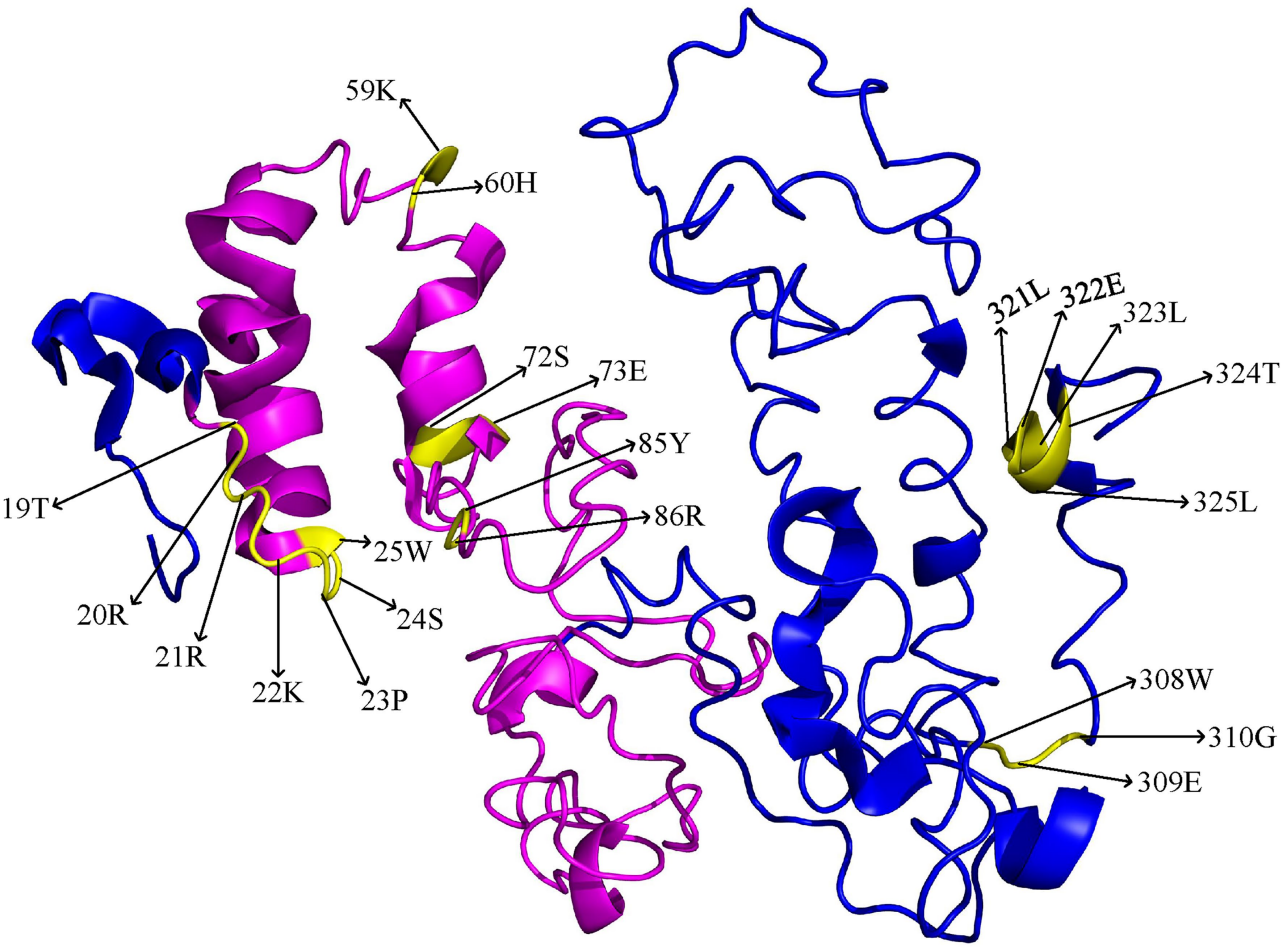
3D model of the *BnaA06BZR1* protein. The BZR1 domain was highlighted in pink, and the yellow color indicates the specific amino acid sites that are involved in functional divergence.

To predict the three-dimensional structure of the BnaBZR proteins, we utilized the representative BnaA06BZR1 protein sequence in the I-TASSER database (Zhang, 2008). The normalized C-score for the predicted model was > −3, suggesting the best template for the BnaBZR1 protein 3-D structure prediction. As shown in Figure 3, we found that the 3D structure of the BnaA06BZR1 contains nine *α*-helicases and one *β*-strands, in which the BZR1 domain has only four *α*-helicases. Furthermore, important amino acid sites, which take part in the functional divergence, were also displayed on the three-dimensional structure of the BnaA06BZR1. Out of 21 amino acid residues, only 14 were dispersed on the BZR1 domain, and the remaining eight sites were spread on the C-terminal region of the BnaA06BZR1, which indicates the BZR1 domain might be more vulnerable to positive selection during the evolution of the *BnaBZR* gene family. Additionally, amino acids residues 19T and 20R that are present in both *ϴ*I and *ϴ*II functional divergence were located on the N-terminal BZR domain, indicating the essential role of these sites in the evolution of the *B. napus BZR* gene family (Figure 3).

### Multiple Sequence Alignment and Motifs Distribution Analysis of *BnaBZRs*

**Figure 4:**
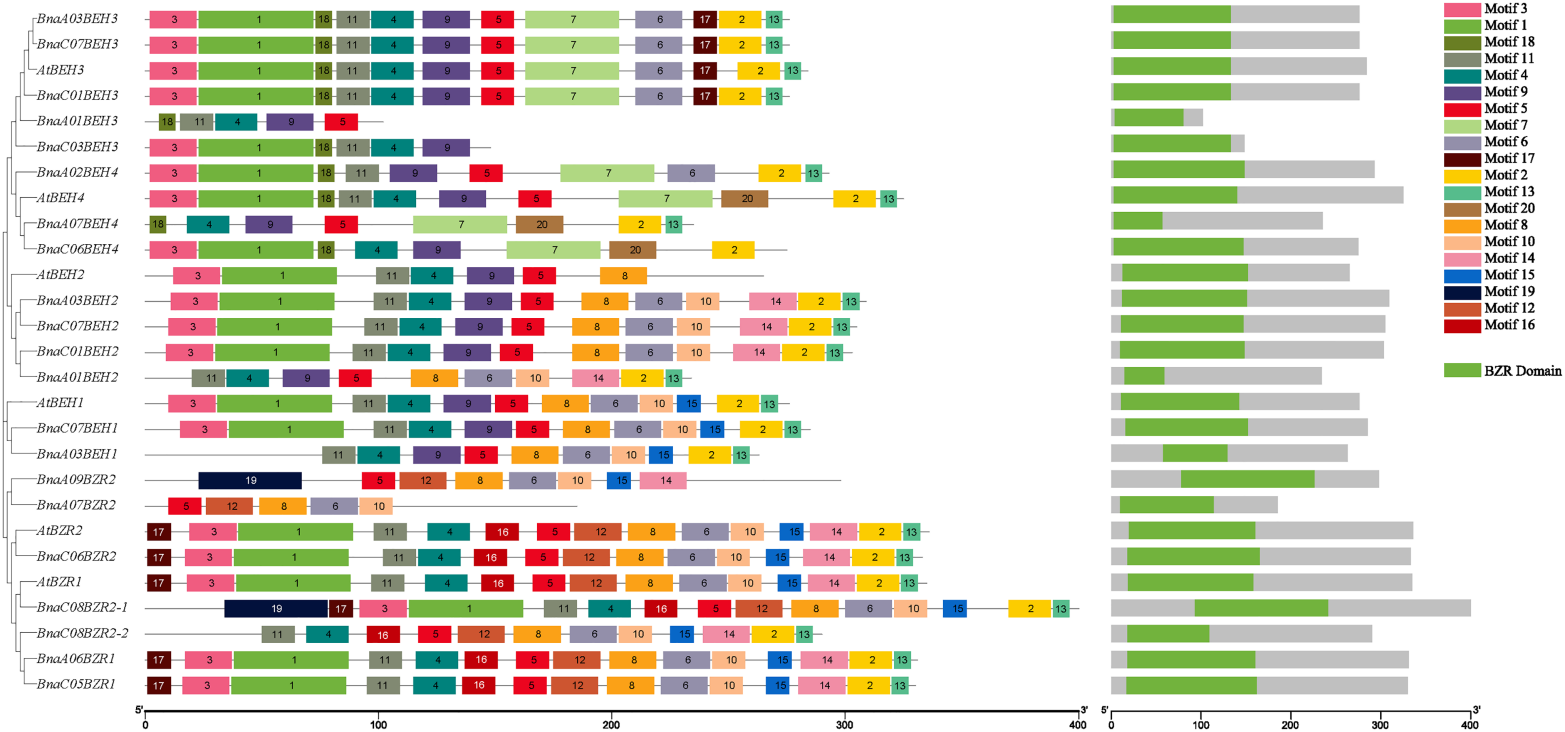
Schematic representation of conserved motifs distribution among *B. napus* and *A. thaliana BZR* gene family. The 20 conserved motifs are indicated by different colored boxes and numbers, the logo of each motif was presented in Supplementary Figure S2. The MEME score for the protein match to the motif model was equals to <1 × 10^−20^. The schematic illustration of BZR domain at the N-terminal regions of the *BnaBZRs* was a set of motifs.

The full-length 42 coding sequences of the BZR proteins from *B. oleracea*, *B. rapa*, *A. thaliana*, and *B. napus* were employed to perform the multiple sequence alignment analysis to predict the highly conserved amino acids sites and examine the structural features of the *BnaBZR* gene family (Supplementary Figure S1). Based on the alignment, we found that six *AtBZRs* showed the highest percent amino acid homology with *BnaBZRs* (Supplementary Table S2). Furthermore, a highly conserved domain BRASSINAZOLE RESISTANT1 (BZR1) was identified in the N-terminal region of the *BnaBZRs* and other denoted plant species (Supplementary Figure S1). In the previous studies, it has been reported that the N-terminal BZR1 domain contains the serine-rich phosphorylation site and participates in modulating the expression of targeted genes to promote various plant physiological processes, such as reproduction, senescence, cell elongation, and cell division (Clouse et al., 1996; Ye et al., 2010). Overall, the domain distribution in *B. napus* and *A. thaliana* was similar (Figure 4). To further classify the diversification of the *B. napus BZR* gene family, we built a graph presenting 20 putative motifs and their distribution on *BnaBZRs* (Figure 4). Our results showed that all *BnaBZRs* contain five to 15 highly conserved motifs within a length range from eight to 50 amino acids. In which motif, 1, 3, 4, 5, and 11 encoded a BZR1 domain (Figure 4). The consensus sequences of these motifs were also shown in (Supplementary Figure S2). All *BnaBZRs* contain motif 5, except for *BnaC03BEH3* and *BnaC06BEH4*, while other remaining motifs existed in some but not in all members (Figure 4). For instance, motif 8 and 10 were only present in group I, II, and III, whereas motif 7 and 18 are only found in group IV, except for *BnaC03BEH3* and *BnaA01BEH3* lacking motif 7. Furthermore, motif 19 was only detected in *BnaC08BZR2-1* and *BnaA09BZR2*. In contrast, motif 20 was only found in *AtBEH4*, *BnaA07BEH4,* and *BnaC06BEH4*. Motif 12 and 16 were only present in group I, except for *BnaA09BZR2* and *BnaA07BZR2*. However, the structure and motif distribution within the same group were similar, suggesting similar biological functions among the same group (Figure 4).

### Chromosomal Location and Genome Duplication Analysis of *BnaBZRs*

**Figure 5:**
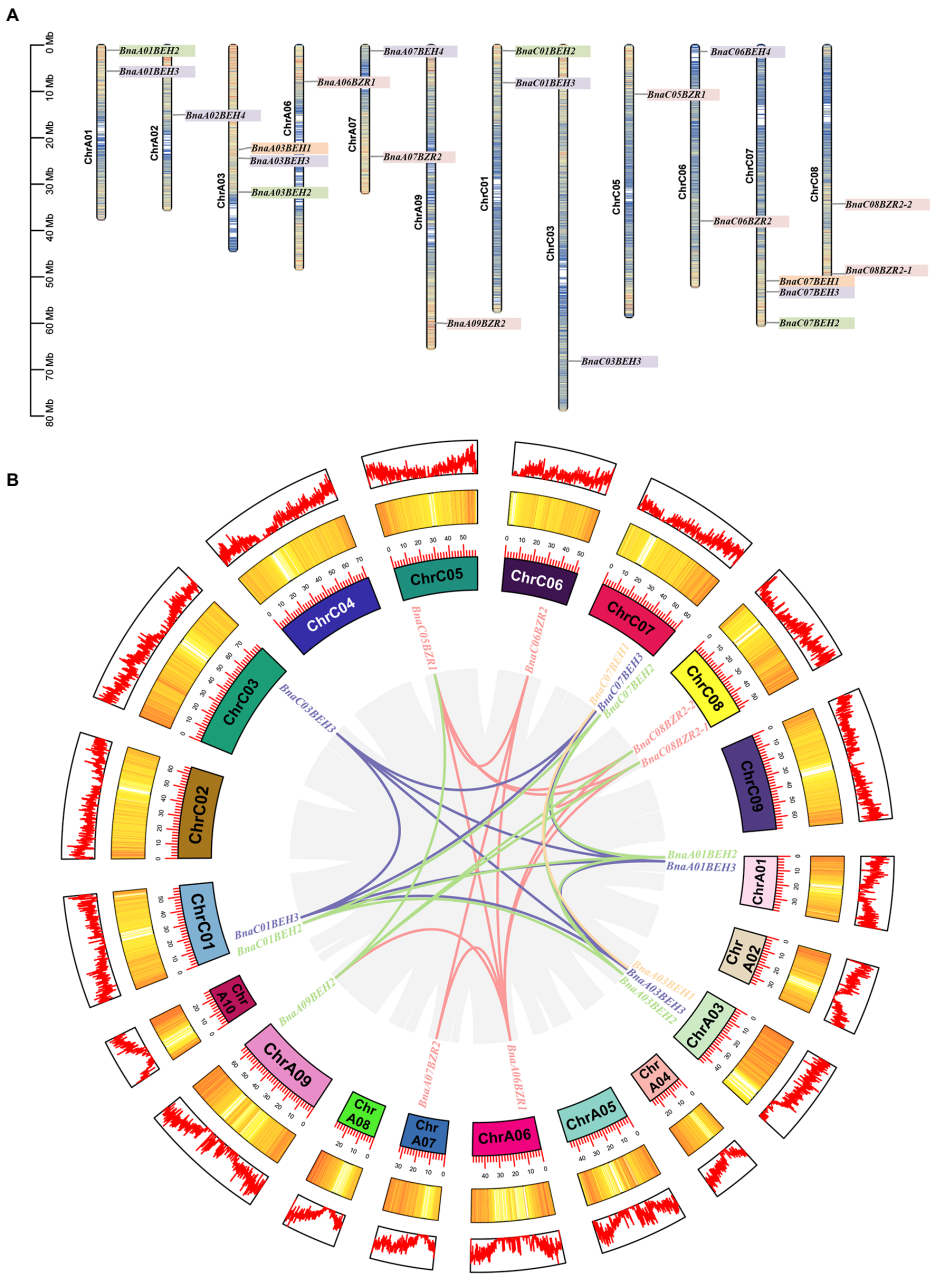
Chromosomal location and genome duplication of *BnaBZRs.* **(A)** Distribution of *BnaBZRs* on *B. napus* chromosomes. Genes from the same group are indicated by the same highlighted color. **(B)** Synteny analysis of *BnaBZRs* in *B. napus.* Lines with same color belong to the same *BnaBZR* gene subfamily indicate segmental duplication in *B. napus* chromosomes. Gray lines in the background indicated as *B. napus* genome synteny blocks. Number of each *B. napus* chromosome is represented as different colored box with the scale size is in Kb, and the outer circle represents the gene density profile of each chromosome.

**Figure 6:**
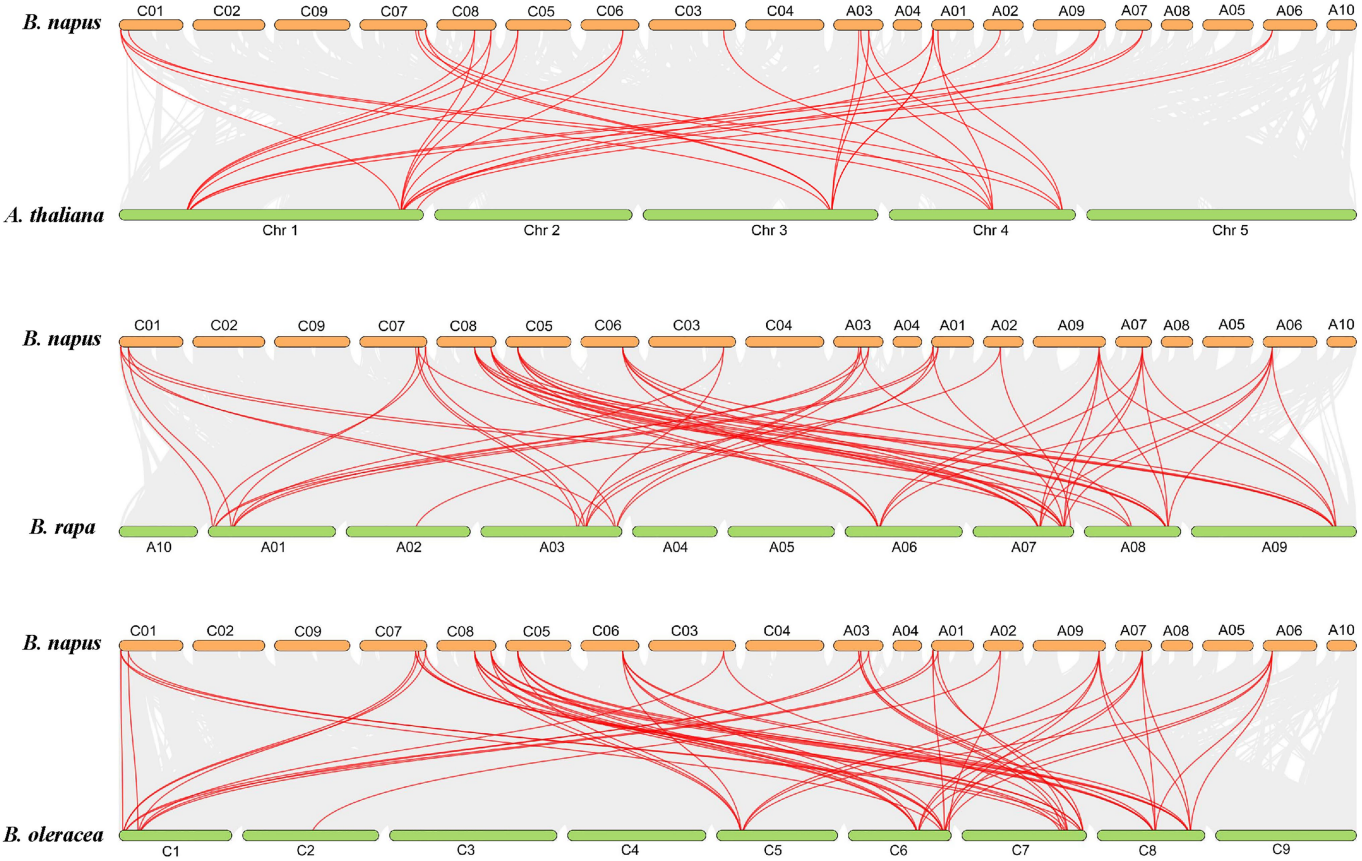
Colinear gene pair analysis of *BnaBZRs* in *B. napus* in comparison with *A. thaliana*, *B. rapa,* and *B. oleracea.* The red lines indicate the collinear *BZR* gene pairs, and the gray lines indicate the collinear pairs within in the *A. thaliana*, *B. napus*, *B. rapa*, and *B. oleracea* genome.

To predict the distribution of *BnaBZRs* in *B. napus* chromosomes, we mapped the 21 *BnaBZR* members into the 19 *B. napus* chromosomes (Figure 5A). Based on their physical position, we found that members of the *BnaBZR* have distributed unevenly between *B. napus* chromosomes. In which chromosome, A07, A01, CO1, C06, and C08 contain two members of *BnaBZR*. Chromosomes A03 and C07 hold three members of the *BnaBZR*, while chromosomes A02, A06, A09, C03, and C05 have only one *BnaBZR* gene (Figure 5A). Furthermore, the MCScanX tool was also implemented to reveal the duplication of *BnaBZRs* in *B. napus*; 29 segmental duplication pairs were identified using this method. However, no tandem duplication pair was detected, implying that segmental duplication events played the central driving role in the functional divergence of *BnaBZRs* during *B. napus* genome duplication (Figure 5B). Moreover, genome duplication variation between four *Brassicaceae* species *A. thaliana, B. napus, B. oleracea,* and *B. rapa* was also investigated (Figure 6). The results show that *B. napus* contains both orthologous and paralogous copies of *BZR* genes. For instance, six *AtBZR* genes (*AT1G19350, AT1G75080, AT3G50750, AT4G36780, AT4G18890*, and *AT1G78700*) have two, five, two, four, five, and three orthologous copies in the *B. napus* genome, respectively (Supplementary Table S3). Moreover, the orthologous copies of *BZR* genes from the *B. napus* ancestor were also predicted. A total of 12 and 13 *BZR* genes from *B. rapa* and *B. oleracea* showed a syntenic relationship with *BnaBZRs*, respectively. These orthologous copies might be the reason for the distribution and functional diversification of the *B. napus BZR* gene family. In addition, we also measured the nonsynonymous (Ka) and synonymous (Ks) ratios for each pair of duplicated BnaBZR proteins (Supplementary Table S4). The results showed that all BnaBZR pairs contain the Ks/Ka ratio < 1, suggesting that BnaBZRs practiced a robust purifying selection (Supplementary Figure S3). However, there are some limitations in calculating the Ka/Ks ratio of the BnaBZRs, because the members of the BnaBZR have minute variations in their sequences, which may bring Ka/Ks value to lower than 1 (Nielsen and Yang, 1998; Nekrutenko et al., 2002). Therefore, we used the site models to predict the selection pressure on single amino acid codons (Yang, 2007; Supplementary Table S5). Model MO is one ratio model, which presumes the same ratio ɯ at all amino acid sites. In our study, we predicted the ratio value ɯ = 0.228 in M0, suggesting the strong purifying selection was the main reason for the evolution of the *BnaBZRs*. Furthermore, we also compared the model M0 and M3 to predict d*N*/d*S* ratio differentiation among codon sites. Interestingly, the log-likelihood 2∆InL was statistically significant (*p* < 0.01), indicating *BnaBZR* experienced immense selective pressure across different sites. Additionally, the models M7 (beta) and M8 (beta + ɯ > 1) were also compared, which considered a very stringent test of positive selection (Anisimova et al., 2001), to predict whether positive selection involves promoting divergence of *BZR* gene family in *B. napus*. However, no positive selection sites were identified. The results from this analysis indicate that the *BnaBZR* gene family underwent divergent selective pressure during duplication (Supplementary Table S5).

### Protein–Protein Interaction Analysis of *BZRs*

**Figure 7:**
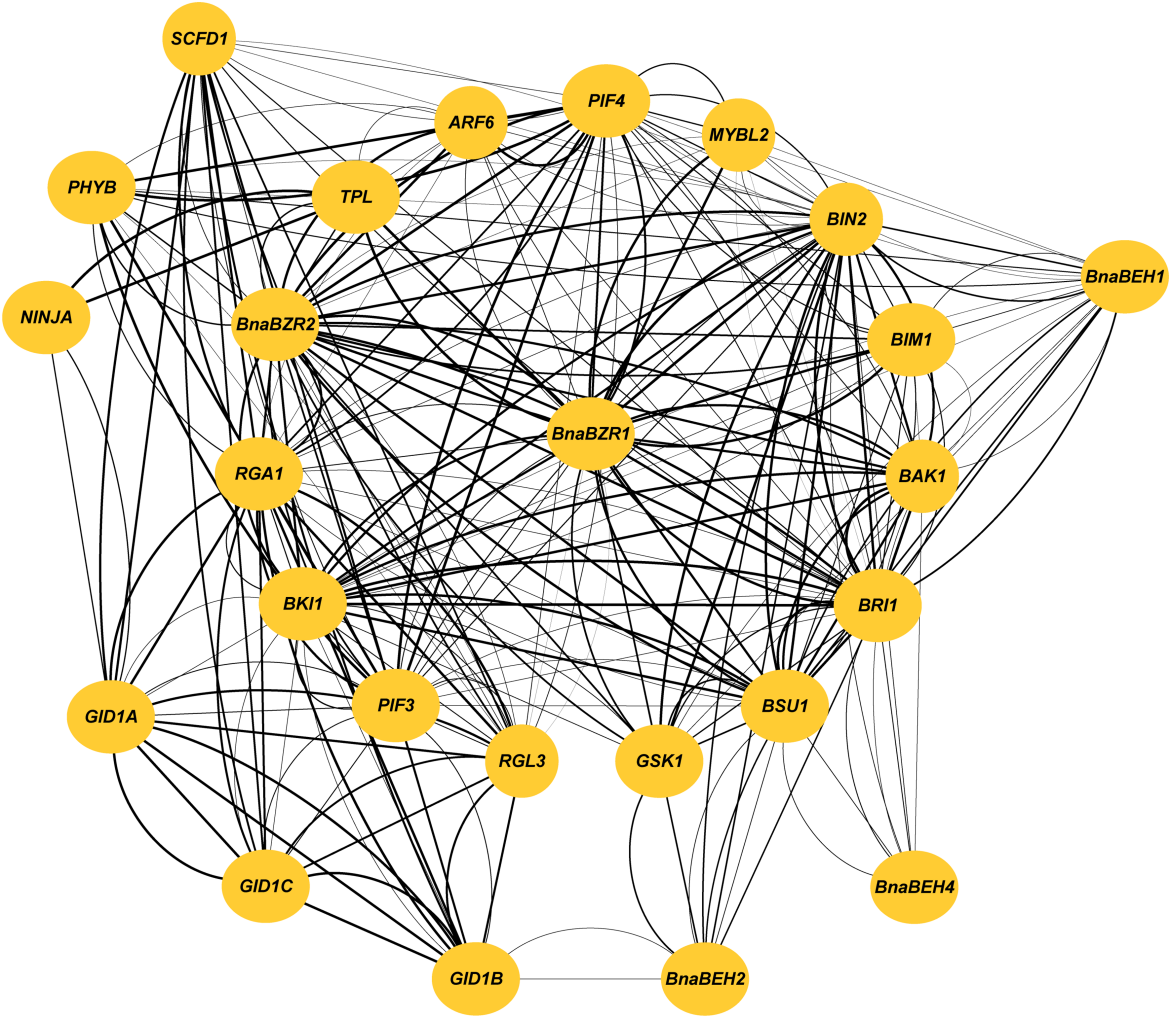
Protein interaction analysis of the BnaBZRs according to the *Arabidopsis* orthologs. Thicker lines represent the stronger interaction.

Protein interaction study provides an essential insight to examine gene function in a systematic study (Pellegrini et al., 2004). Our analysis predicted that the BnaBZR proteins are highly homologous to the *A. thaliana* BZR proteins. Therefore, to further examine the interacting relationship of the BnaBZRs with its targeting partners, we constructed a network using the AtBZRs orthologs. As shown in Figure 7, we found that the members in the group I interact with more targeted proteins than others. BES1-INTERACTING MYC-LIKE1 (BIM1), BRI1 KINASE INHIBITOR 1 (BKI1), ARABIDOPSIS MYB-LIKE 2 (MYBL2), and PHYTOCHROME INTERACTING FACTOR 4 (PIF4) were predicted to bind with BnaBZR1, BnaBZR2, and BnaBEH1, while PHYTOCHROME B (PHYB) was only found to interact with BnaBZR2 and BnaBEH1 (Supplementary Table S6). However, the biological functions associated with their interaction are still unclear. In contrast, BRASSINOSTEROID-INSENSITIVE 2 (BIN2), BRASSINOSTEROID-INSENSITIVE 1 (BRI1), and BRI1 SUPPRESSOR 1 (BSU1) only interact with BnaBZR1 BnaBZR2, BnaBEH1, BnaBEH2, and BnaBEH4, whereas PHYTOCHROME INTERACTING FACTOR 3 (PIF3), REPRESSOR OF GA (RGA1), RGA-LIKE PROTEIN 3 (RGL3), and AUXIN RESPONSE FACTOR 6 (ARF6) are found to interacts with BnaBZR1 and BnaBZR2. In previous studies, it has been reported that the interaction of BZR1 with ARF6 and PIF mediates auxin-induced plant growth in response to environmental stresses. However, under constant stress conditions, gibberellin negative regulator RGA binds with the BZR1 to inhibit its transcriptional activity (Oh et al., 2014). Additionally, SLEEPY1 (SLY1) and TOPLESS (TPL) are only found to interact with BnaBZR2. Similarly, GA INSENSITIVE DWARF1B (GID1B) shows only interaction with BnaBEH2, which indicates the possible role of BnaBEH2 in regulating BR and GAs crosstalk in response to stresses. It is also worth noting that BnaBEH3 displays no interaction with other proteins (Figure 7). This analysis provides significant clues to understand the relation of the BnaBZRs with unknown proteins in different signaling pathways.

### *cis*-Acting Elemental Analysis

To further examine the individual role of *BnaBZR* members and how these genes respond to different stresses, we investigated the *cis*-regulatory elements in the promoter sequences of the *BnaBZRs*. It has been proposed that the homologs genes with similar roles might consist of the same *cis*-regulatory elements in their promoter regions. As shown in Supplementary Figure S4, a set of *cis*-elements belonging to the plant development, stress and defense, and hormonal response-related elements were detected (Supplementary Table S7), but their numbers and distribution were uneven in all *BnaBZRs* (Supplementary Figure S4A). For instance, *BnaC01BEH3*, *BnaA03BEH2*, and *BnaC07BEH2* contained the highest number of development-related, hormone-responsive, and light-responsive *cis*-elements, respectively. In contrast, *BnaA06BZR1* contains many stress and defense-related *cis*-regulatory elements (Supplementary Figure S4B). Nearly, all of the *BnaBZRs* hold the basic promoter elements including, CAAT-box and TATA-box. However, several core *cis*-elements were only being seen in a few *BnaBZRs*. For instance, Sp1 (light-responsive *cis*-element) and TGA-box (*cis*-element take part in ABA responsiveness) were only found in *BnaA06BZR1* and *BnaC05BZR1*. A-Box (*cis*-element involved in the development process), CCGTCC-Box (*cis*-acting element activates the meristem development), and DRE-core (regulates drought and cold-responsive gene expression) were detected in *BnaC05BZR2*, *BnaC08BZR2*-1, and *BnaC06BZR2*. Furthermore, OCT (*cis*-element involved in cell proliferation), GCN4-motif (*cis*-element involved in nitrogen-related response), chs-CMA2a (light-responsive element), and DRE1 (regulates drought and cold-responsive gene expression) were detected in *BnaC06BZR2*, *BnaC07BEH1*, and *BnaA01BEH2*, respectively. GATT-motif, SARE (*cis*-element involved in salicylic acid response), 3-AF1 binding site (light-responsive *cis*-element), CARE (involved in ABA response), and LAMP-element were absent in all *BnaBZRs* except in *BnaC08BZR2-2*, *BnaA07BZR2*, *BnaA03BEH1*, *BnaC07BEH2*, and *BnaC07BEH3*, respectively. Similarly, AT1-motif, Box-III, motif-I, and CTAG-motif were only found in *BnaC01BHE3.* In addition, CAG-motif, ATC-motif, and dOCT (*cis*-element involved in cell cycle responses) were detected in *BnaC01BEH2* and *BnaC03BEH3*. Overall, light-responsive *cis*-elements were the most abundant in all *BnaBZRs* promoters, which indicates the transcriptional activity of the *BnaBZRs* might be induced by light. Additionally, *cis*-elements related to hormone responses, such as auxin (AUX), gibberellins (GA), ABA, and salicylic acid (SA), were predicted, in which *cis*-element involved in ABA responses were the most common in all *BnaBZRs*. Moreover, stress and defense responsive *cis*-elements, including ARE (anaerobic regulatory element), WUN-motif (regulates wound-related response), LTR (associated with cold response), and STRE, which activated by the several stress condition, especially heat shock, nutrient deficiency, and osmotic stress were also predicted. Results from this analysis showed that the *BnaBZRs* contain various kinds of development and stress-related responsive elements, indicating that the *BnaBZRs* might control plant physiology in response to several phytohormones and environmental stresses.

### *BnaBZRs* Expression Profiling in Different Tissues

**Figure 8:**
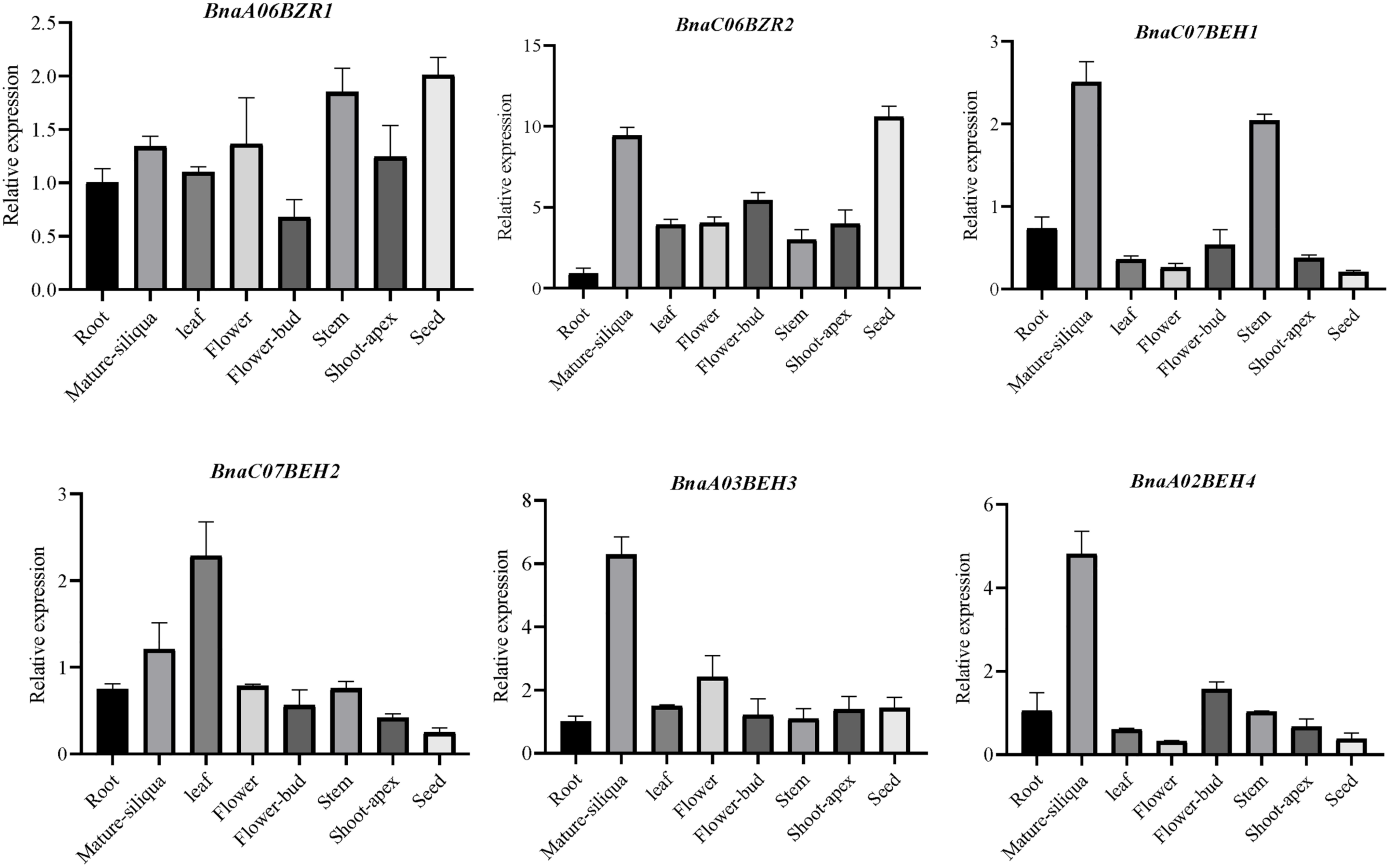
Organ-specific expression pattern analysis of selected *BnaBZRs* in different tissues. The expression abundance of the specified *BnaBZRs* was adjusted with respect to the *B. napus Actin* gene. Tissues names are shown on the *x*-axis. The error bars on the *y*-axis represent the standard error of three independent biological replicates (listed in Supplementary Table S8.2).

To further explore the potential functions of the *BnaBZR* gene family, we study their expression patterns in cotyledon, leaf, silique, root, petal, bud, lower stem, sepal, vegetative rosette, pollen, upper stem, middle stem, seed, and filament of the *B. napus* variant Zhongshuang 11 (ZS11), by using the transcriptomic data available in BnTIR database http://yanglab.hzau.edu.cn/BnTIR. As shown in Supplementary Figure S5, 17 members of the *BnaBZR* gene family exhibited higher expression in sepal, petal, seed, lower stem, middle stem, upper stem, root, vegetative rosette, cotyledon, filament, and siliqua, while no or mild expression was observed in pollen and bud (Supplementary Table S8.1). In detail, pairs of homologs genes *BnaC08BZR2-1/BnaC08BZR2-2*, *BnaA09BZR2,* and *BnaA07BZR2/BnaC06BZR2* showed higher expression in sepal, petal, and seed. In contrast, *BnaA01BEH2*, *BnaA03BEH3*, *BnaC07BEH3*, and *BnaA02BEH4* higher expressions were observed in cotyledon and siliqua. Nevertheless, *BnaA06BZR1* and *BnaC05BZR1* display relatively lower expression in four tissues (root, sepal, upper stem, and vegetative rosette; Supplementary Figure S5). To further validate the organ-specific expression of the *BnaBZRs*, the relative expression pattern of the six putative *BnaBZRs* was determined in eight tissues of *B. napus* cultivar Zhongshuang 11 (ZS11) including mature-silique, shoot-apex, seed, leaf, flower-bud, flower, stem, and root by qRT-PCR. As shown in Figure 8, two of six *BnaBZRs* (*BnaA06BZR1* and *BnaC06BZR2*) were consistently expressed in all selected tissues, except for *BnaA06BZR1,* which shows relatively lower expression in flower-bud. Additionally, the increased expression level of the *BnaCO6BZR2*, *BnaC07BEH1*, *BnaA03BEH3,* and *BnaA02BEH4* was mainly observed in the mature siliqua (Figure 8, Supplementary Table S8.2). In contrast, *BnaC07BEH2* was expressed highly in the leaf. Combined with transcriptomic data, the expression pattern of the *BnaC07BEH1*, *BnaA03BEH3*, and *BnaA02BEH4* was also observed in the siliqua. However, the correlation test between the two datasets showed a non-significant association (*r* = 0.435, *p* = 0.713). The non-significant association between transcriptome data and qRT-PCR might be related to harvesting of siliqua at 38 and 44 days after flowering, respectively. Therefore, the fold change should not be expected to have same for both datasets. Overall, most members of the *BnaBZRs* expressed differently in the plant tissues, suggesting the key roles of *BnaBZRs* in various tissues during plant development.

### Expression Analysis of the *BnaBZRs* During Different Stress Conditions

**Figure 9:**
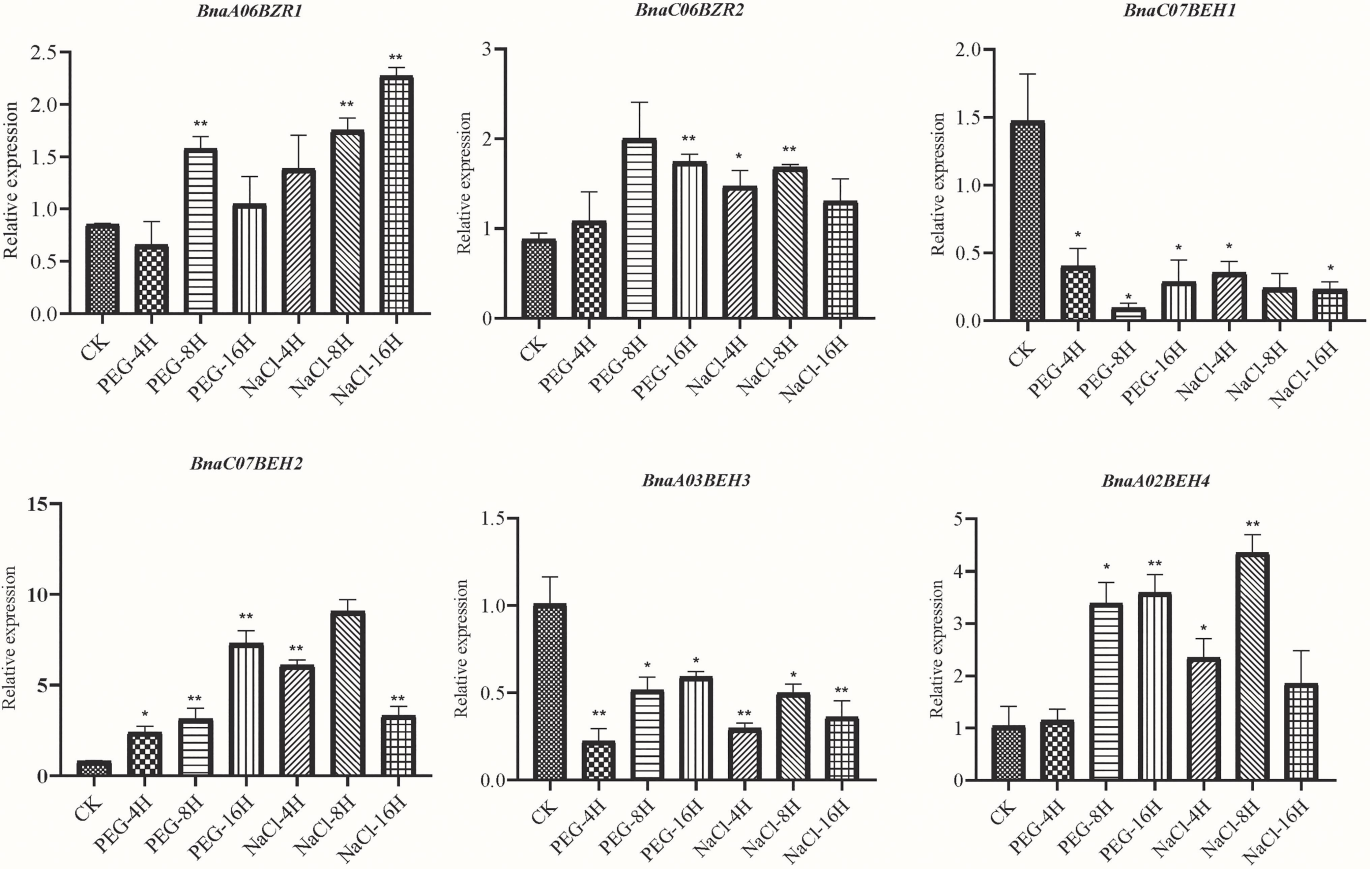
The relative expression analysis of the *BnaBZRs* under different treatments. The expression abundance of the selected *BnaBZRs* was controlled with respect to the reference gene (*Actin*). The *x*-axis corresponds to different treatments, and values on the *y*-axis are denoted as the mean ± SE of three biological replicates (listed in Supplementary Table S9.2). Asterisk on vertical bar represent a significant difference at ^*^p < 0.05 and ^**^p < 0.01.

To gain further insights into the *BnaBZR* gene family in response to the environmental stresses, we investigated their expression levels in drought, salinity, cold, and *S. sclerotiorum* stresses using publicly available RNA-seq data (CRA001775 and SRP311601). As shown in Supplementary Figure S6A, we found that the expression level of group I and group II was downregulated after 2 h of the drought treatment (Supplementary Table S9.1). Nevertheless, the expression pattern of the *BnaA03BEH3* and *BnaC06BEH4* were induced. In contrast, the expression pattern of the four *BnaBZRs* (*BnaA07BZR2*, *BnaC06BZR2*, *BnaA03BEH3*, and *BnaC07BEH3*) was peaked at 8 h of drought treatment. Under salinity treatment, the expression pattern of the *BnaA03BEH3* and *BnaC07BEH3* was first induced at 4 h but then reduced in 24 h of salinity treatment. Furthermore, under cold treatment, the RNA transcripts of the group IV members *BnaC03BEH3*, *BnaA02BEH4*, and *BnaA07BEH4* were upregulated during 24 h of cold treatment. However, *BnaA07BEH4* and *BnaC06BEH4*-induced expression was only observed in *S. sclerotiorum* infection (Supplementary Figure S6B). To elucidate the functions of the *BnaBZRs* in abiotic stresses, we monitored their expression changes by qRT-PCR in leaves of *B. napus* cultivar (ZS11) treated with different stresses, such as drought and salinity (Figure 9). On the whole, the expression patterns of the *BnaBZRs* were significantly changed during 8–16 h of drought and salt treatment (Supplementary Table S9.2). During salt treatment, *BnaA06BZR1*, *BnaC06BZR2*, and *BnaA02BEH4* were dramatically induced at 8 h and reduced at 16 h, while *BnaC07BEH1, BnaA03BEH3,* and *BnaC07BEH2* showed downregulation in their expression patterns, except for *BnaC07BEH2,* which showed upregulated expression at 16 h of drought treatment. Furthermore, expression patterns of the *BnaC07BEH2* and *BnaA02BEH4* were significantly increased at 8 h but then reduced at 16 h of NaCl treatment, indicating the expression patterns of the *BnaC07BEH2* and *BnaA02BEH4* were induced in more than one stress. In our, organ-specific expression analysis, we observed the different expression patterns of the *BnaBZRs* in various tissues (Supplementary Figure S5, Figure 8). Therefore, it is possible that the *BnaBZR* gene family in *B. napus* responded to drought, salinity, cold, and *S. sclerotiorum* stress might play a diverse regulatory function in various tissues.

### Prediction of Potential microRNA Targets in *BnaBZR* Gene Family

MicroRNAs regulate gene expression by cleaving the mRNA of the targeted genes in response to stresses (Song et al., 2019). Identifying the miRNA-targeted genes employing the computational approaches is vital for predicting the transcriptional regulatory role of the gene family. Therefore, to understand the transcription network and post-transcriptional regulation of the *BnaBZRs*, we examine the potential miRNA target sites using the psRNATarget database. Out of the 21 *BnaBZRs*, only nine genes were targeted by 12 novel bna-miRNA, with an average of one to two targets per bna-miRNA (Table 3). The two members of group I were predicted to be targeted by three novel bna-miRNA (bna-miR395, bna-miR171, and bna-miR170), whereas six members of group IV were targeted by the bna-miR158, bna-miR164, bna-miR159, bna-miRN273, and bna-miRN292. In addition, only one member of group II (*BnaC07BEH1*) was targeted by the bna-miRN291 and bna-miRN285. However, except for the bna-miRN285/*BnaC07BEH1* and bna-miR164/*BnaA02BEH4*, the majority of the bna-miRNA-targeted genes was expected to be silenced by the cleavage inhibition. Furthermore, we have also predicted the UPE value, which is within 8.94–21.43, needed to unpair the secondary structure around the target site. Nevertheless, most of the bna-miRNA identified in this study, such as bna-miRNA395 (Huang et al., 2010; Zhang et al., 2013a), bna-miR171 (Jian et al., 2016), and bna-miR159, are stimulated by the environmental cues, suggesting that different bna-miRNAs direct post-transcriptional regulation of some of the members of the *BnaBZR* gene family during biotic and abiotic stresses.

**Table 3:** miRNAs targeting *BnaBZRs.*

miRNA_Acc.	Gene symbol	Target_Acc.	Target start	Target end	miRNA aligned fragment	Alignment length	*E*-value	UPE
Bna-miR395	*BnaC08BZR2-1*	*BnaC08G0463100ZS*	1,067	1,087	CUGAAGUGUUUGGGGGAACUC	21	3	21.43
Bna-miR171	*BnaC08BZR2-1*	*BnaC08G0463100ZS*	502	522	UGAUUGAGCCGUGCCAAUAUC	21	5	8.95
	*BnaC08BZR2-2*	*BnaC08G0253000ZS*	139	159	UGAUUGAGCCGCGCCAAUAUC	21	5	9.69
Bna-miR170	*BnaC08BZR2-2*	*BnaC08G0253000ZS*	139	159	UGAUUGAGCCGCGUCAAUAUC	21	4	9.69
Bna-miRN291	*BnaC07BEH1*	*BnaC07G0389100ZS*	111	132	AAAUAUUUUAGGUGAUGUGGCA	23	4	16.75
Bna-miRN285			609	630	UUCGGGGAUUUCUGUGUAGGCU	23	4.5	13.79
Bna-miR158	*BnaC01BEH3*	*BnaC01G0121000ZS*	723	743	UUUCCAAAUGUAGACAAAGCA	21	4.5	11.91
Bna-miR164	*BnaA02BEH4*	*BnaA02G0233100ZS*	274	294	UGGAGAAGCAGGGCACGUGCA	21	5	13.98
Bna-miR164i			364	384	UGGAGAAGCAGAGCACGUGCA	21	5	13.98
Bna-miR159	*BnaA01BEH3*	*BnaA01G0099000ZS*	96	116	UUUGGAUUGAAGGGAGCUCUA	21	4.5	14.52
	*BnaC03BEH3*	*BnaC03G0707200ZS*	297	317	UUUGGAUUGAAGGGAGCUCUA	21	5	16.23
Bna-miRN273	*BnaC06BEH4*	*BnaC06g39100D*	174	194	GGUUCGUUGAUCUGGCCGGAC	21	4.5	12.53
Bna-miRN292	*BnaA07BEH4*	*BnaA07g34330D*	420	440	GGGCUUCGUGGGAUUAGUCGG	21	4.5	12.25
	*BnaC06BEH4*	*BnaC06g39100D*	540	560	GGGCUUCGUGGGAUUAGUCGG	21	4.5	8.94

### GO Enrichment Analysis and Sequence Polymorphism of *BnaBZRs* in Core Germplasm of *Brassica napus*

It is well reported that a family of transcriptional factors BZR interacts with several sequences to regulate plant tolerance to environmental cues. To further examine the biological role of *BnaBZRs*, we conducted the gene ontology (GO) enrichment analysis. Our results showed that a total of 43 GO terms were categorized into three groups, BP (biological process), CC (cellular component), and MF (molecular function; Supplementary Table S10). Among BP category, all *BnaBZRs* were within the “metabolic processes (GO:1901360, GO:0006725, GO:0034641, GO:0046483, GO:0006807),” “biosynthetic processes (GO:1901576, GO:0034645, GO:0009059, GO:0009058),” “gene expression (GO:0010467),” and “cellular process (GO:0050794),” whereas *BnaC05G0163000ZS*, *BnaA06G0135100ZS*, *BnaA02G0233100ZS*, *BnaA01G0099000ZS*, and *BnaC03G0707200ZS* might be involved in plant tolerance to stresses (GO:0006950, GO:0006952, GO:0006955, GO:0009607, and GO:0009617). Under the CC category, all *BnaBZRs* were in the nucleus (GO:0005634), while some of the genes, such as *BnaA09G0609500ZS*, *BnaC08G0463100ZS*, *BnaC05G0163000ZS*, *BnaA06G0135100ZS*, *BnaC06G0273700ZS*, *BnaA07G0248700ZS*, *BnaC08G0253000ZS*, *BnaA03G0416600ZS*, and *BnaC07G0389100ZS,* were also predicted in the cytosol (GO:0005829) and cytoplasm (GO:0005737) category, respectively. Additionally, in the MF category, all *BnaBZRs* were significantly enriched in binding (GO:0003677, GO:0003700, and GO:1901363) together with “organic cyclic compound binding (GO:0097159)” (Supplementary Figure S7). Results from this analysis displayed the diverse roles of the *BnaBZRs* in various biological processes, mainly in cellular and metabolic processes and biological process regulation.

**Figure 10:**
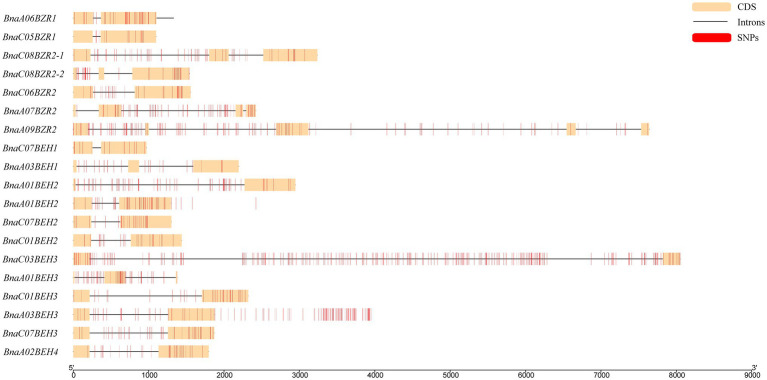
SNPs distribution on coding and non-coding regions of the *BnaBZRs.* The yellow-colored block represents as the coding sequence, whereas the black lines indicate as the introns, and red-colored block represents as SNP.

To locate the sequence polymorphism of the *BnaBZRs*, we used the sequencing data of 159 accessions of rape seed from the *B. napus* genomic browser [BnPIR (Song et al., 2020, 2021)]. On the whole, 55.5% of SNPs were predicted in the *BnaBZR* gene family (Supplementary Table S11). However, the SNP percentage frequency on each subfamily was dissimilar. For instance, group I, II, III, and IV hold an average of 49, 18, 39.2, and 64.8 SNPs, respectively. For *BnaA07BEH4* and *BnaC06BEH*, no SNP was found. Meanwhile, we also observed that the SNPs density of the *BnaBZRs* on CC chromosomes was relatively higher than the AA chromosomes. Furthermore, a detailed SNPs distribution on the coding and non-coding regions of the *BnaBZR* gene family was also shown (Figure 10), which display that the distribution of SNPs in introns is varied between 9.5–52.8%, in which the highest number of SNPs (224) was found in the intron region of *BnaC03BEH3*. However, in exons, the distribution of SNPs was 8.5–20.14%, whereas the lowest number of SNPs (4) was found in the exon region of *BnaA03BEH1*. Results from this analysis indicate that the sequence variation in the members of the *BnaBZRs* might be associated with their differential expression pattern under different conditions.

## Discussion

Under the external stimulus, plants modulate their responses to adapt to the complex environment. It has been reported that the BR regulates plant physiology by downstream components to stimulate environmental adaptation (Xie et al., 2011; Miyaji et al., 2014). These downstream components were transcriptional factors of BR signaling BZR1 and BZR2, which regulate plant growth and tolerance to stresses by integrating with a wide variety of BR-responsive genes. In recent years, due to the advancement in high-throughput sequencing, the BZR gene family in many economically important crops, such as Rice, Maize, *Brassica rapa, Glycine max,* Tomato, *Eucalyptus grandis*, and *Petunia hybrid* (Tong et al., 2012; Verhoef et al., 2013; Liu et al., 2014; Saha et al., 2015; Zhang et al., 2016; Fan et al., 2018; Manoli et al., 2018), have been isolated and examined (Table 1). These studies confirmed the roles of the *BZR* genes in plant biological processes and reported the possible function in stress conditions. For instance, *SiBZR1* might regulate BR-signaling in response to salt stress in tomato (Jia et al., 2021). In contrast, the overaccumulation of the *AtBZR1* in *Glycine max* induces the seed count per siliqua (Zhang et al., 2016). Furthermore, few studies have also reported the transcriptional response of BR signaling during environmental stresses in *B. napus* (Kagale et al., 2007; Sahni et al., 2016). For instance, overexpression of the *A. thaliana* BR biosynthetic gene in *B. napus* enhances drought tolerance phenotype, which is likely mediated by BZR transcriptional factors (Sahni et al., 2016). Moreover, there is only one study that helped to understand the cross-genome exploration of *BZR* origin between *B. napus* and other plant species (Song et al., 2018). However, there is a lack of studies regarding the structural features, functional divergence, and stress-resistant related response of the BZR members in the *B. napus*, which gives us an excellent opportunity to inspect the *B. napus BZR* gene family and analyze their features including evolutionary relationship, structure, protein evolution and functional divergence, protein–protein interaction, miRNA prediction, *cis*-elements, and expression profile in different organs and response to abiotic stresses. These findings provide an overview of the *BZR* genes structural features, evolution as well as important insights regarding the potential unknown functions of the *BnaBZRs.*

A total of 21 *BnaBZRs* were identified, which are separated into four groups based on *A. thaliana BZR* homologs (Figure 1). However, the numbers of *BnaBZRs* were higher than the *B. oleracea*, *A. thaliana*, and *B. rapa*. This variation in gene numbers might be due to the larger genome or genome duplication during the evolution of the *B. napus*. Group I and group II, III are the sister groups, while members in group IV are distantly associated with other groups. In terms of amino acid similarity, all members in the groups contain the highly conserved N-terminal BZR1 domain, but the gene structure and motif composition display discrepancies in family members. For instance, members in group IV contain only one intron, while most members in group I, II, and III contain two introns, although the exon distribution among family members is different (Figure 2). Moreover, group IV lacks motif 8, 10, and 14 (Figure 4), indicating that the genes from the same subfamily might function differently among other groups. The diversity among gene sub-families is mainly driven by the mutations in the specific amino acid site (Lynch and Conery, 2000; Ha et al., 2009). To identify the functional divergence between the *BnaBZRs* groups, *ϴ*I and *ϴ*II functional divergence analysis was performed. Results from this analysis showed that the total of 21 *ϴ*I and three *ϴ*II functional divergence sites were predicted in three subfamily (Table 2), suggesting that the *ϴ*I functional divergence is dominant. Additionally, two amino acid sites, 19T and 20R, were predicted in both *ϴ*I and *ϴ*II. These sites might be responsible for diversification in evolutionary rates and physiochemical properties. Interestingly, we observed that *ϴ*I and *ϴ*II functional divergence sites were mainly distributed on the N-terminal BZR1 domain (Figure 3), suggesting that the *BnaBZR* gene family might experience varied selection pressures during duplication events. However, the insufficiency of significant variations in functional divergence among *BnaBZR* gene family pairs indicates the similar roles of their family members. Furthermore, synteny analysis predicts that the members in group IV have similar segmental duplication patterns in group I, II, and III, suggesting that the sub-functionalization or non-functionalization may include during the evolution of the *B. napus BZR* gene family. Nevertheless, segmental duplication appears to contribute more to *BZR* gene family expansion than tandem duplication during duplication events (Figure 5B). *Brassica napus* is an allopolyploid (AACC) crop, originated from the natural hybridization of the *B. oleracea* and *B. rapa* (Chalhoub et al., 2014), which were diverged approximately 20–40 years ago from the common ancestor *A. thaliana*. To study the genetic relationship among them, we performed the collinearity analysis, which indicates the 49, 62, and 62 colinear gene pairs between *B. napus/A. thaliana*, *B. napus/B. rapa*, and *B. napus/B. oleracea*, respectively (Figure 6; Supplementary Table S3).

Additionally, we investigate that *BnaBZRs* expressed ubiquitously in sepal, petal, seed, lower stem, middle stem, upper stem, root, vegetative rosette, cotyledon, filament, and siliqua wall (Supplementary Figure S5; Figure 8). According to the previous studies, the BR signaling pathway is needed to regulate the transition from vegetative to reproductive growth (Gallego-Bartolomé et al., 2012; Li et al., 2012; Oh et al., 2012; Zhiponova et al., 2013; Fridman et al., 2014; Vragović et al., 2015), indicating that the members of the *BnaBZR*s might mediate *B. napus* development by interacting with various TFs of different signaling pathways. In our protein interaction analysis, the members in the *BnaBZR* gene family show a strong association with hormonal and stress-induced transcriptional factors (Figure 7). For instance, BnaBZR1, BnaBZR2, and BnaBEH1 interact with MYBL2, PHYB, PIF3, PIF4, RGA1, RGL3, SLY1, and GID1B, which function in response to anthocyanin biosynthesis, light, gibberellins (GA), ABA, salt, and drought (Somers and Quail, 1995; Más et al., 2000; Mahmood et al., 2016). Based on these results, we speculate that the *BnaBZR*s might regulate plant physiology in response to stresses. However, the molecular mechanism underlying these interactions needs verification. To further examine the roles of the *BnaBZR*s during stresses, the different expression patterns of *BnaBZRs* were observed against salinity, drought, cold, and *S. sclerotiorum* stresses (Supplementary Figure S6; Figure 9). The results are consistent with the previous findings that reported BZR genes in other species. For instance, in *B. rapa*, the expression patterns of six *BrBZRs* display higher expression in response to salt stress, whereas 11 BrBZRs showed induced expression in response to drought stress (Saha et al., 2015). However, in maize, members of the *BZR* gene family exhibit reduced expression during heat stress (Manoli et al., 2018), while increased expression was observed during salinity stress. Besides this in RNA-seq analysis, we predicted the induced expression patterns of the *BnaBEH3* and *BnaBEH4* genes under *S. sclerotiorum* infection (Supplementary Figure S6B). Notably, the promoter regions of the *BnaBZRs* were predicted to contain the biotic stress-responsive *cis*-core elements (Supplementary Table S7), suggesting the possible role of the *BnaBZR* TFs in response to biotic stresses.

In addition, to examine the *BnaBZRs* regulatory pathways, we predicted the miRNAs targeted genes, which gives an important insight into the genes regulation role under stresses. In the *B. napus*, a total of 376 miRNAs had been identified (Zhang et al., 2019); however, only 88 novel miRNAs are predicted to show important roles in *B. napus* (Palatnik et al., 2007; Alonso-Peral et al., 2010; Shen et al., 2014). In this study, we observed that the nine members of the *BnaBZR*s were targeted by different miRNAs. In particular, *BnaBEH3* and *BnaBEH4,* whose expression was significantly regulated under drought and salinity stress, show an oblivious interaction with bna-miR158, bna-miR164, bna-miR159, and bna-miRN273 (Table 3), which are reported to exhibit genes regulations at the post-transcriptional level in response to various stresses (Wang et al., 2012a; Zhao et al., 2012; Huang et al., 2013; Zhang et al., 2018). Furthermore, our GO analysis revealed that BP terms, such as response to biotic stimulus, immune response, response to the bacterium, defense response, and response to stresses, were present in *BnaBEH3* and *BnaBEH4* (Supplementary Figure S7). Therefore, our results suggest the prominent regulatory role of group III and group IV genes during abiotic and biotic stresses. However, the functional mechanism of these genes in *B. napus* still needs to be investigated, as this will help us understand the functional divergence of the *BnaBZR* gene family and enable further dissection of BR-signaling in *B. napus.*

## Conclusion

In our study, a total of 21 *BnaBZRs* were recognized in *B. napus* genomic sequence, which was clustered into the four groups based upon the phylogenetic relationship among *B. oleracea*, *B. rapa,* and *A. thaliana* orthologs. Our protein sequence similarity analysis displayed that all members of the *BnaBZRs* are closely related to six *A. thaliana BZR* genes, which indicate similar structural features. Genome duplication and synteny analysis revealed that segmental duplication was the main reason for *BZR* genes expansion through purifying selection in *B. napus*. Furthermore, protein–protein interaction miRNA target prediction, *cis*-regulatory elements, Gene Ontology, and qRT-PCR analysis of *BnaBZRs* under salt and drought stress exhibit an obvious divergence between subgroups. Overall, results from this study provide valuable information on evolutionary characteristics and potential functions of the *BnaBZR*s in abiotic and biotic stresses, which lay a foundation for future work in modulating stress tolerance and *B. napus* development.

## Data Availability Statement

The datasets presented in this study can be found in online repositories. The names of the repository/repositories and accession number(s) can be found in the article/Supplementary Material.

## Author Contributions

RS conceived and planned the experiments and wrote the manuscript. RG, LL, and YS extracted the RNA and performed the qRT-PCR analysis. K-MZ and JW analyzed the data. X-LT revised and supervised the manuscript. All authors contributed to the article and approved the submitted version.

## Funding

This study was funded by the Jiangsu Agriculture Science and Technology Innovation Fund (CX(21)2009).

## Conflict of Interest

The authors declare that the research was conducted in the absence of any commercial or financial relationships that could be construed as a potential conflict of interest.

## Publisher’s Note

All claims expressed in this article are solely those of the authors and do not necessarily represent those of their affiliated organizations, or those of the publisher, the editors and the reviewers. Any product that may be evaluated in this article, or claim that may be made by its manufacturer, is not guaranteed or endorsed by the publisher.

## Footnotes

^1^ https://www.genoscope.cns.fr/brassicanapus

^2^ http://cbi.hzau.edu.cn/bnapus

^3^ http://www.arabidopsis.org

^4^ http://brassicadb.cn

^5^ https://prosite.expasy.org

^6^ http://pfam.xfam.org/search/sequence

^7^ http://web.expasy.org

^8^ http://www.csbio.sjtu.edu.cn/bioinf/plant-multi

^9^ http://itol.embl.de/

^10^ http://gsds.cbi.pku.edu.cn

^11^ http://meme-suite.org/tools/meme

^12^ http://selecton.tau.ac.il

^13^ https://zhanggroup.org//I-TASSER/

^14^ https://pymol.org/

^15^ https://string-db.org/

^16^ http://www.mirbase.org/

^17^ http://structuralbiology.cau.edu.cn/PNRD/index.php

^18^ https://www.zhaolab.org/psRNATarget/

^19^ http://bioinformatics.psb.ugent.be/webtools/plantcare/html

## References

[ref1] Alonso-Peral M. M. Li J. Li Y. Allen R. S. Schnippenkoetter W. Ohms S.. (2010). The microRNA159-regulated GAMYB-like genes inhibit growth and promote programmed cell death in *Arabidopsis*. Plant Physiol. 154, 757–771. doi: 10.1104/pp.110.160630, PMID: 20699403 PMC2949021

[ref2] Anisimova M. Bielawski J. P. Yang Z. (2001). Accuracy and power of the likelihood ratio test in detecting adaptive molecular evolution. Mol. Biol. Evol. 18, 1585–1592. doi: 10.1093/oxfordjournals.molbev.a003945, PMID: 11470850

[ref3] Bailey T. L. Elkan C. (1994). Fitting a mixture model by expectation maximization to discover motifs in bipolymers. Proc. Int. Conf. Intell. Syst. Mol. Biol. 2, 28–36. doi: 10.2139/ssrn.3705225, PMID: 7584402

[ref4] Cao X. Khaliq A. Lu S. Xie M. Ma Z. Mao J.. (2020). Genome-wide identification and characterization of the BES1 gene family in apple (*Malus domestica*). Plant Biol. 22, 723–733. doi: 10.1111/plb.13109, PMID: 32141196

[ref5] Chalhoub B. Denoeud F. Liu S. Parkin I. A. Tang H. Wang X.. (2014). Early allopolyploid evolution in the post-Neolithic *Brassica napus* oilseed genome. Science 345, 950–953. doi: 10.1126/science.1253435, PMID: 25146293

[ref6] Chen C. Chen H. Zhang Y. Thomas H. R. Frank M. H. He Y.. (2020). TBtools: an integrative toolkit developed for interactive analyses of big biological data. Mol. Plant 13, 1194–1202. doi: 10.1101/289660, PMID: 32585190

[ref9] Chen J. Nolan T. M. Ye H. Zhang M. Tong H. Xin P.. (2017). *Arabidopsis* WRKY46, WRKY54, and WRKY70 transcription factors are involved in brassinosteroid-regulated plant growth and drought responses. Plant Cell 29, 1425–1439. doi: 10.1105/tpc.17.00364, PMID: 28576847 PMC5502465

[ref7] Chen L.-G. Gao Z. Zhao Z. Liu X. Li Y. Zhang Y.. (2019a). BZR1 family transcription factors function redundantly and indispensably in BR signaling but exhibit BRI1-independent function in regulating anther development in Arabidopsis. Mol. Plant 12, 1408–1415. doi: 10.1016/j.molp.2019.06.006, PMID: 31229643

[ref8] Chen W. Lv M. Wang Y. Wang P.-A. Cui Y. Li M.. (2019b). BES1 is activated by EMS1-TPD1-SERK1/2-mediated signaling to control tapetum development in *Arabidopsis thaliana*. Nat. Commun. 10:4164. doi: 10.1038/s41467-019-12118-4, PMID: 31519953 PMC6744560

[ref10] Chen X. Wu X. Qiu S. Zheng H. Lu Y. Peng J.. (2021). Genome-wide identification and expression profiling of the BZR transcription factor gene family in *Nicotiana benthamiana*. Int. J. Mol. Sci. 22:10379. doi: 10.3390/ijms221910379, PMID: 34638720 PMC8508657

[ref11] Chinchilla D. Shan L. He P. de Vries S. Kemmerling B. (2009). One for all: the receptor-associated kinase BAK1. Trends Plant Sci. 14, 535–541. doi: 10.1016/j.tplants.2009.08.002, PMID: 19748302 PMC4391746

[ref12] Chou K.-C. Shen H.-B. (2010). Plant-mPLoc: a top-down strategy to augment the power for predicting plant protein subcellular localization. PLoS One 5:e11335. doi: 10.1371/journal.pone.0011335, PMID: 20596258 PMC2893129

[ref13] Clouse S. D. Langford M. McMorris T. C. (1996). A brassinosteroid-insensitive mutant in *Arabidopsis thaliana* exhibits multiple defects in growth and development. Plant Physiol. 111, 671–678. doi: 10.1104/pp.111.3.671, PMID: 8754677 PMC157882

[ref14] Cui X.-Y. Gao Y. Guo J. Yu T.-F. Zheng W.-J. Liu Y.-W.. (2019). BES/BZR transcription factor TaBZR2 positively regulates drought responses by activation of TaGST1. Plant Physiol. 180, 605–620. doi: 10.1104/pp.19.00100, PMID: 30842265 PMC6501075

[ref15] Dai X. Zhao P. X. (2011). psRNATarget: a plant small RNA target analysis server. Nucleic Acids Res. 39, W155–W159. doi: 10.1093/nar/gkr319, PMID: 21622958 PMC3125753

[ref16] De Castro E. Sigrist C. J. Gattiker A. Bulliard V. Langendijk-Genevaux P. S. Gasteiger E.. (2006). ScanProsite: detection of PROSITE signature matches and ProRule-associated functional and structural residues in proteins. Nucleic Acids Res. 34, W362–W365. doi: 10.1093/nar/gkl124, PMID: 16845026 PMC1538847

[ref17] Ding L. Guo X. Wang K. Pang H. Liu Y. Yang Q.. (2021). Genome-wide analysis of BES1/BZR1 transcription factors and their responses to osmotic stress in *Ammopiptanthus nanus*. J. For. Res. 26, 127–135. doi: 10.1080/13416979.2020.1867293

[ref18] Fan C. Guo G. Yan H. Qiu Z. Liu Q. Zeng B. (2018). Characterization of Brassinazole resistant (BZR) gene family and stress induced expression in *Eucalyptus grandis*. Physiol. Mol. Biol. Plants 24, 821–831. doi: 10.1007/s12298-018-0543-2, PMID: 30150857 PMC6103948

[ref19] Fridman Y. Elkouby L. Holland N. Vragović K. Elbaum R. Savaldi-Goldstein S. (2014). Root growth is modulated by differential hormonal sensitivity in neighboring cells. Genes Dev. 28, 912–920. doi: 10.1101/gad.239335.114, PMID: 24736847 PMC4003282

[ref20] Friedt W. Tu J. Fu T. (2018). “Academic and economic importance of *Brassica napus* rapeseed,” in The *Brassica napus* Genome. eds. Friedt W. Rod S. Boulos C. (Cham: Springer), 1–20.

[ref21] Gallego-Bartolomé J. Minguet E. G. Grau-Enguix F. Abbas M. Locascio A. Thomas S. G.. (2012). Molecular mechanism for the interaction between gibberellin and brassinosteroid signaling pathways in *Arabidopsis*. Proc. Natl. Acad. Sci. U. S. A. 109, 13446–13451. doi: 10.1073/pnas.1119992109, PMID: 22847438 PMC3421204

[ref22] Gasteiger E. Hoogland C. Gattiker A. Wilkins M. R. Appel R. D. Bairoch A. (2005). “Protein identification and analysis tools on the ExPASy Server,” in The Proteomics Protocols Handbook. Springer Protocols Handbooks. ed. Walker J. M. (Humana Press), 571–607.

[ref23] Glazebrook J. (2001). Genes controlling expression of defense responses in *Arabidopsis*—2001 status. Curr. Opin. Plant Biol. 4, 301–308. doi: 10.1016/s1369-5266(00)00177-1, PMID: 11418339

[ref24] Gu X. Vander Velden K. (2002). DIVERGE: phylogeny-based analysis for functional–structural divergence of a protein family. Bioinformatics 18, 500–501. doi: 10.1093/bioinformatics/18.3.500, PMID: 11934757

[ref25] Gu X. Zou Y. Su Z. Huang W. Zhou Z. Arendsee Z.. (2013). An update of DIVERGE software for functional divergence analysis of protein family. Mol. Biol. Evol. 30, 1713–1719. doi: 10.1093/molbev/mst069, PMID: 23589455

[ref26] Guo X. Lu P. Wang Y. Cai X. Wang X. Zhou Z.. (2017). Genome-wide identification and expression analysis of the gene family encoding Brassinazole resistant transcription factors in cotton. Cotton Sci. 29, 415–427.

[ref27] Ha M. Kim E.-D. Chen Z. J. (2009). Duplicate genes increase expression diversity in closely related species and allopolyploids. Proc. Natl. Acad. Sci. U. S. A. 106, 2295–2300. doi: 10.1073/pnas.0807350106, PMID: 19168631 PMC2650150

[ref28] Hatzig S. V. Nuppenau J.-N. Snowdon R. J. Schießl S. V. (2018). Drought stress has transgenerational effects on seeds and seedlings in winter oilseed rape (*Brassica napus* L.). BMC Plant Biol. 18:297. doi: 10.1186/s12870-018-1531-y, PMID: 30470194 PMC6251133

[ref29] He J.-X. Gendron J. M. Sun Y. Gampala S. S. Gendron N. Sun C. Q.. (2005). BZR1 is a transcriptional repressor with dual roles in brassinosteroid homeostasis and growth responses. Science 307, 1634–1638. doi: 10.1126/science.1107580, PMID: 15681342 PMC2925132

[ref30] He J.-X. Gendron J. M. Yang Y. Li J. Wang Z.-Y. (2002). The GSK3-like kinase BIN2 phosphorylates and destabilizes BZR1, a positive regulator of the brassinosteroid signaling pathway in *Arabidopsis*. Proc. Natl. Acad. Sci. 99, 10185–10190. doi: 10.1073/pnas.152342599, PMID: 12114546 PMC126645

[ref31] He K. Gou X. Yuan T. Lin H. Asami T. Yoshida S.. (2007). BAK1 and BKK1 regulate brassinosteroid-dependent growth and brassinosteroid-independent cell-death pathways. Curr. Biol. 17, 1109–1115. doi: 10.1016/j.cub.2007.05.036, PMID: 17600708

[ref32] Hu B. Jin J. Guo A.-Y. Zhang H. Luo J. Gao G. (2015). GSDS 2.0: an upgraded gene feature visualization server. Bioinformatics 31, 1296–1297. doi: 10.1093/bioinformatics/btu817, PMID: 25504850 PMC4393523

[ref33] Huang D. Koh C. Feurtado J. A. Tsang E. W. Cutler A. J. (2013). MicroRNAs and their putative targets in *Brassica napus* seed maturation. BMC Genomics 14:140. doi: 10.1186/1471-2164-14-140, PMID: 23448243 PMC3602245

[ref34] Huang D. W. Sherman B. T. Lempicki R. A. (2009a). Bioinformatics enrichment tools: paths toward the comprehensive functional analysis of large gene lists. Nucleic Acids Res. 37, 1–13. doi: 10.1093/nar/gkn923, PMID: 19033363 PMC2615629

[ref35] Huang D. W. Sherman B. T. Lempicki R. A. (2009b). Systematic and integrative analysis of large gene lists using DAVID bioinformatics resources. Nat. Protoc. 4, 44–57. doi: 10.1038/nprot.2008.211, PMID: 19131956

[ref36] Huang S. Q. Xiang A. L. Che L. L. Chen S. Li H. Song J. B.. (2010). A set of miRNAs from *Brassica napus* in response to sulphate deficiency and cadmium stress. Plant Biotechnol. J. 8, 887–899. doi: 10.1111/j.1467-7652.2010.00517.x, PMID: 20444207

[ref38] Jia C. Zhao S. Bao T. Zhao P. Peng K. Guo Q.. (2021). Tomato BZR/BES transcription factor SlBZR1 positively regulates BR signaling and salt stress tolerance in tomato and *Arabidopsis*. Plant Sci. 302:110719. doi: 10.1016/j.plantsci.2020.110719, PMID: 33288025

[ref37] Jia D. Chen L. G. Yin G. Yang X. Gao Z. Guo Y.. (2020). Brassinosteroids regulate outer ovule integument growth in part via the control of INNER NO OUTER by BRASSINOZOLE-RESISTANT family transcription factors. J. Integr. Plant Biol. 62, 1093–1111. doi: 10.1111/jipb.12915, PMID: 32009278

[ref39] Jian H. Wang J. Wang T. Wei L. Li J. Liu L. (2016). Identification of rapeseed microRNAs involved in early stage seed germination under salt and drought stresses. Front. Plant Sci. 7:658. doi: 10.3389/fpls.2016.00658, PMID: 27242859 PMC4865509

[ref40] Jiang S. Li S. Liu X. Wen B. Wang N. Zhang R.. (2021). Genome-wide identification and characterization of the MdBZR1 gene family in apple and their roles in improvement of drought tolerance. Sci. Hortic. 288:110359. doi: 10.1016/j.scienta.2021.110359

[ref41] Kagale S. Divi U. K. Krochko J. E. Keller W. A. Krishna P. (2007). Brassinosteroid confers tolerance in *Arabidopsis thaliana* and *Brassica napus* to a range of abiotic stresses. Planta 225, 353–364. doi: 10.1007/s00425-006-0361-6, PMID: 16906434

[ref42] Kang S. Yang F. Li L. Chen H. Chen S. Zhang J. (2015). The *Arabidopsis* transcription factor BRASSINOSTEROID INSENSITIVE1-ETHYL METHANESULFONATE-SUPPRESSOR1 is a direct substrate of MITOGEN-ACTIVATED PROTEIN KINASE6 and regulates immunity. Plant Physiol. 167, 1076–1086. doi: 10.1104/pp.114.250985, PMID: 25609555 PMC4348755

[ref43] Kesawat M. S. Kherawat B. S. Singh A. Dey P. Kabi M. Debnath D.. (2021). Genome-wide identification and characterization of the Brassinazole-resistant (BZR) gene family and its expression in the various developmental stage and stress conditions in wheat (*Triticum aestivum* L.). Int. J. Mol. Sci. 22:8743. doi: 10.3390/ijms22168743 34445448 PMC8395832

[ref44] Kim T.-W. Guan S. Burlingame A. L. Wang Z.-Y. (2011). The CDG1 kinase mediates brassinosteroid signal transduction from BRI1 receptor kinase to BSU1 phosphatase and GSK3-like kinase BIN2. Mol. Cell 43, 561–571. doi: 10.1016/j.molcel.2011.05.037, PMID: 21855796 PMC3206214

[ref45] Kim Y. Song J.-H. Park S.-U. Jeong Y.-S. Kim S.-H. (2017). Brassinosteroid-induced transcriptional repression and dephosphorylation-dependent protein degradation negatively regulate BIN2-interacting AIF2 (a BR signaling-negative regulator) bHLH transcription factor. Plant Cell Physiol. 58, 227–239. doi: 10.1093/pcp/pcw223, PMID: 28069895

[ref46] Kozomara A. Birgaoanu M. Griffiths-Jones S. (2019). miRBase: from microRNA sequences to function. Nucleic Acids Res. 47, D155–D162. doi: 10.1093/nar/gky1141, PMID: 30423142 PMC6323917

[ref47] Lachowiec J. Mason G. A. Schultz K. Queitsch C. (2018). Redundancy, feedback, and robustness in the *Arabidopsis thaliana* BZR/BEH gene family. Front. Genet. 9:523. doi: 10.3389/fgene.2018.00523, PMID: 30542366 PMC6277886

[ref48] Lescot M. Déhais P. Thijs G. Marchal K. Moreau Y. Van de Peer Y.. (2002). PlantCARE, a database of plant cis-acting regulatory elements and a portal to tools for in silico analysis of promoter sequences. Nucleic Acids Res. 30, 325–327. doi: 10.1093/nar/30.1.325, PMID: 11752327 PMC99092

[ref49] Letunic I. Bork P. (2021). Interactive Tree Of Life (iTOL) v5: an online tool for phylogenetic tree display and annotation. Nucleic Acids Res. 49, W293–W296. doi: 10.1093/nar/gkab301, PMID: 33885785 PMC8265157

[ref56] Li H. Ye K. Shi Y. Cheng J. Zhang X. Yang S. (2017). BZR1 positively regulates freezing tolerance via CBF-dependent and CBF-independent pathways in Arabidopsis. Mol. Plant 10, 545–559. doi: 10.1016/j.molp.2017.01.004, PMID: 28089951

[ref50] Li J. Chory J. (1999). Brassinosteroid actions in plants. J. Exp. Bot. 50, 275–282. doi: 10.1093/jexbot/50.332.275

[ref53] Li J. Nam K. H. Vafeados D. Chory J. (2001). BIN2, a new brassinosteroid-insensitive locus in *Arabidopsis*. Plant Physiol. 127, 14–22. doi: 10.1104/pp.127.1.14, PMID: 11553730 PMC117958

[ref55] Li L. Ye H. Guo H. Yin Y. (2010). *Arabidopsis* IWS1 interacts with transcription factor BES1 and is involved in plant steroid hormone brassinosteroid regulated gene expression. Proc. Natl. Acad. Sci. U. S. A. 107, 3918–3923. doi: 10.1073/pnas.0909198107, PMID: 20139304 PMC2840484

[ref51] Li Q.-F. He J.-X. (2016). BZR1 interacts with HY5 to mediate brassinosteroid-and light-regulated cotyledon opening in Arabidopsis in darkness. Mol. Plant 9, 113–125. doi: 10.1016/j.molp.2015.08.014, PMID: 26363272

[ref52] Li Q.-F. Lu J. Yu J.-W. Zhang C.-Q. He J.-X. Liu Q.-Q. (2018). The brassinosteroid-regulated transcription factors BZR1/BES1 function as a coordinator in multisignal-regulated plant growth. Biochim. Biophys. Acta 1861, 561–571. doi: 10.1016/j.bbagrm.2018.04.003 29673687

[ref54] Li Q.-F. Wang C. Jiang L. Li S. Sun S. S. He J.-X. (2012). An interaction between BZR1 and DELLAs mediates direct signaling crosstalk between brassinosteroids and gibberellins in *Arabidopsis*. Sci. Signal. 5:ra72. doi: 10.1126/scisignal.2002908, PMID: 23033541

[ref57] Lin E. Zhuang H. Yu J. Liu X. Huang H. Zhu M.. (2020). Genome survey of Chinese fir (*Cunninghamia lanceolata*): identification of genomic SSRs and demonstration of their utility in genetic diversity analysis. Sci. Rep. 10, 4698–4612. doi: 10.1038/s41598-020-61611-0, PMID: 32170167 PMC7070021

[ref58] Liu D. Cui Y. Zhao Z. Li S. Liang D. Wang C.. (2021). Genome-wide identification and characterization of the BES/BZR gene family in wheat and foxtail millet. BMC Genomics 22:682. doi: 10.1186/s12864-021-08002-5, PMID: 34548036 PMC8456565

[ref59] Liu L. Jia C. Zhang M. Chen D. Chen S. Guo R.. (2014). Ectopic expression of a BZR1-1D transcription factor in brassinosteroid signalling enhances carotenoid accumulation and fruit quality attributes in tomato. Plant Biotechnol. J. 12, 105–115. doi: 10.1111/pbi.12121, PMID: 24102834

[ref60] Livak K. J. Schmittgen T. D. (2001). Analysis of relative gene expression data using real-time quantitative PCR and the 2−ΔΔCT method. Methods 25, 402–408. doi: 10.1006/meth.2001.1262 11846609

[ref61] Lynch M. Conery J. S. (2000). The evolutionary fate and consequences of duplicate genes. Science 290, 1151–1155. doi: 10.1126/science.290.5494.1151, PMID: 11073452

[ref62] Mahmood K. Xu Z. El-Kereamy A. Casaretto J. A. Rothstein S. J. (2016). The *Arabidopsis* transcription factor ANAC032 represses anthocyanin biosynthesis in response to high sucrose and oxidative and abiotic stresses. Front. Plant Sci. 7:1548. doi: 10.3389/fpls.2016.01548, PMID: 27790239 PMC5063858

[ref63] Manoli A. Trevisan S. Quaggiotti S. Varotto S. (2018). Identification and characterization of the BZR transcription factor family and its expression in response to abiotic stresses in Zea mays L. Plant Growth Regul. 84, 423–436. doi: 10.1007/s10725-017-0350-8

[ref64] Más P. Devlin P. F. Panda S. Kay S. A. (2000). Functional interaction of phytochrome B and cryptochrome 2. Nature 408, 207–211. doi: 10.1046/j.1365-313x.1999.t01-1-00599.x, PMID: 11089975

[ref65] Mi H. Muruganujan A. Ebert D. Huang X. Thomas P. D. (2019). PANTHER version 14: more genomes, a new PANTHER GO-slim and improvements in enrichment analysis tools. Nucleic Acids Res. 47, D419–D426. doi: 10.1093/nar/gky1038, PMID: 30407594 PMC6323939

[ref66] Mistry J. Chuguransky S. Williams L. Qureshi M. Salazar G. A. Sonnhammer E. L.. (2021). Pfam: the protein families database in 2021. Nucleic Acids Res. 49, D412–D419. doi: 10.6019/tol.pfam_fams-t.2018.00001.1, PMID: 33125078 PMC7779014

[ref67] Miyaji T. Yamagami A. Kume N. Sakuta M. Osada H. Asami T.. (2014). Brassinosteroid-related transcription factor BIL1/BZR1 increases plant resistance to insect feeding. Biosci. Biotechnol. Biochem. 78, 960–968. doi: 10.1080/09168451.2014.910093, PMID: 25036120

[ref68] Nekrutenko A. Makova K. D. Li W.-H. (2002). The KA/KS ratio test for assessing the protein-coding potential of genomic regions: an empirical and simulation study. Genome Res. 12, 198–202. doi: 10.1101/gr.200901, PMID: 11779845 PMC155263

[ref69] Nielsen R. Yang Z. (1998). Likelihood models for detecting positively selected amino acid sites and applications to the HIV-1 envelope gene. Genetics 148, 929–936. doi: 10.1093/genetics/148.3.929, PMID: 9539414 PMC1460041

[ref70] Nuruzzaman M. Manimekalai R. Sharoni A. M. Satoh K. Kondoh H. Ooka H.. (2010). Genome-wide analysis of NAC transcription factor family in rice. Gene 465, 30–44. doi: 10.1016/j.gene.2010.06.008 20600702

[ref71] Oh E. Zhu J.-Y. Bai M.-Y. Arenhart R. A. Sun Y. Wang Z.-Y. (2014). Cell elongation is regulated through a central circuit of interacting transcription factors in the Arabidopsis hypocotyl. elife 3:e03031. doi: 10.7554/elife.03031, PMID: 24867218 PMC4075450

[ref72] Oh E. Zhu J.-Y. Wang Z.-Y. (2012). Interaction between BZR1 and PIF4 integrates brassinosteroid and environmental responses. Nat. Cell Biol. 14, 802–809. doi: 10.1038/ncb2545, PMID: 22820378 PMC3703456

[ref73] Palatnik J. F. Wollmann H. Schommer C. Schwab R. Boisbouvier J. Rodriguez R.. (2007). Sequence and expression differences underlie functional specialization of Arabidopsis microRNAs miR159 and miR319. Dev. Cell 13, 115–125. doi: 10.1016/j.devcel.2019.09.016, PMID: 17609114

[ref74] Pellegrini M. Haynor D. Johnson J. M. (2004). Protein interaction networks. Expert Rev. Proteomics 1, 239–249. doi: 10.1586/14789450.1.2.239 15966818

[ref75] Reise S. P. Waller N. G. (2009). Item response theory and clinical measurement. Annu. Rev. Clin. Psychol. 5, 27–48. doi: 10.1146/annurev.clinpsy.032408.153553 18976138

[ref76] Ryu H. Cho H. Bae W. Hwang I. (2014). Control of early seedling development by BES1/TPL/HDA19-mediated epigenetic regulation of ABI3. Nat. Commun. 5, 4138–4111. doi: 10.1038/ncomms5138, PMID: 24938150

[ref77] Sabagh A. E. Hossain A. Barutçular C. Islam M. S. Ratnasekera D. Kumar N.. (2019). Drought and salinity stress management for higher and sustainable canola (‘*Brassica napus*’ L.) production: a critical review. Aust. J. Crop. Sci. 13, 88–97. doi: 10.21475/ajcs.19.13.01.p1284

[ref78] Saha G. Park J.-I. Jung H.-J. Ahmed N. U. Kayum M. A. Kang J.-G.. (2015). Molecular characterization of BZR transcription factor family and abiotic stress induced expression profiling in *Brassica rapa*. Plant Physiol. Biochem. 92, 92–104. doi: 10.1016/j.plaphy.2015.04.013, PMID: 25931321

[ref79] Sahni S. Prasad B. D. Liu Q. Grbic V. Sharpe A. Singh S. P.. (2016). Overexpression of the brassinosteroid biosynthetic gene DWF4 in *Brassica napus* simultaneously increases seed yield and stress tolerance. Sci. Rep. 6, 1–14. doi: 10.1038/srep28298 27324083 PMC4915011

[ref80] Sarwar R. Jiang T. Ding P. Gao Y. Tan X. Zhu K. (2021). Genome-wide analysis and functional characterization of the DELLA gene family associated with stress tolerance in *B. napus*. BMC Plant Biol. 21:286. doi: 10.1186/s12870-021-03054-x, PMID: 34157966 PMC8220683

[ref81] Shen Y. Zhao Q. Zou J. Wang W. Gao Y. Meng J.. (2014). Characterization and expression patterns of small RNAs in synthesized *Brassica hexaploids*. Plant Mol. Biol. 85, 287–299. doi: 10.1007/s11103-014-0185-x, PMID: 24584845

[ref82] Solovyev V. Kosarev P. Seledsov I. Vorobyev D. (2006). Automatic annotation of eukaryotic genes, pseudogenes and promoters. Genome Biol. 7(Suppl 1), S10.1–S10.12. doi: 10.1186/gb-2006-7-s1-s10, PMID: 16925832 PMC1810547

[ref83] Somers D. E. Quail P. H. (1995). Temporal and spatial expression patterns of PHYA and PHYB genes in *Arabidopsis*. Plant J. 7, 413–427. doi: 10.1046/j.1365-313x.1995.7030413.x, PMID: 7757114

[ref84] Song J.-M. Guan Z. Hu J. Guo C. Yang Z. Wang S.. (2020). Eight high-quality genomes reveal pan-genome architecture and ecotype differentiation of *Brassica napus*. Nat. Plants 6, 34–45. doi: 10.1038/s41477-019-0577-7, PMID: 31932676 PMC6965005

[ref86] Song J. M. Liu D. X. Xie W. Z. Yang Z. Guo L. Liu K.. (2021). BnPIR: *Brassica napus* pan-genome information resource for 1689 accessions. Plant Biotechnol. J. 19, 412–414. doi: 10.1111/pbi.13491, PMID: 33068485 PMC7955874

[ref85] Song X. Li Y. Cao X. Qi Y. (2019). MicroRNAs and their regulatory roles in plant–environment interactions. Annu. Rev. Plant Biol. 70, 489–525. doi: 10.1146/annurev-arplant-050718-100334, PMID: 30848930

[ref87] Song X. Ma X. Li C. Hu J. Yang Q. Wang T.. (2018). Comprehensive analyses of the BES1 gene family in *Brassica napus* and examination of their evolutionary pattern in representative species. BMC Genomics 19:346. doi: 10.1186/s12864-018-4744-4 29743014 PMC5944053

[ref88] Stern A. Doron-Faigenboim A. Erez E. Martz E. Bacharach E. Pupko T. (2007). Selecton 2007: advanced models for detecting positive and purifying selection using a Bayesian inference approach. Nucleic Acids Res. 35, W506–W511. doi: 10.1093/nar/gkm382, PMID: 17586822 PMC1933148

[ref89] Su D. Xiang W. Wen L. Lu W. Shi Y. Liu Y.. (2021). Genome-wide identification, characterization and expression analysis of BES1 gene family in tomato. BMC Plant Biol. 21:161. doi: 10.1186/s12870-021-02933-7 33784975 PMC8010994

[ref90] Sun G. Yao T. Feng C. Chen L. Li J. Wang L. (2017). Identification and biocontrol potential of antagonistic bacteria strains against *Sclerotinia sclerotiorum* and their growth-promoting effects on *Brassica napus*. Biol. Control 104, 35–43. doi: 10.1016/j.biocontrol.2016.10.008

[ref91] Swift M. L. (1997). GraphPad prism, data analysis, and scientific graphing. J. Chem. Inf. Comput. Sci. 37, 411–412. doi: 10.1021/ci960402j

[ref92] Szklarczyk D. Gable A. L. Lyon D. Junge A. Wyder S. Huerta-Cepas J.. (2019). STRING v11: protein–protein association networks with increased coverage, supporting functional discovery in genome-wide experimental datasets. Nucleic Acids Res. 47, D607–D613. doi: 10.1093/nar/gky1131, PMID: 30476243 PMC6323986

[ref93] Tong H. Liu L. Jin Y. Du L. Yin Y. Qian Q.. (2012). DWARF AND LOW-TILLERING acts as a direct downstream target of a GSK3/SHAGGY-like kinase to mediate brassinosteroid responses in rice. Plant Cell 24, 2562–2577. doi: 10.1105/tpc.112.097394, PMID: 22685166 PMC3406904

[ref94] Verhoef N. Yokota T. Shibata K. de Boer G.-J. Gerats T. Vandenbussche M.. (2013). Brassinosteroid biosynthesis and signalling in *Petunia hybrida*. J. Exp. Bot. 64, 2435–2448. doi: 10.1093/jxb/ert102, PMID: 23599276 PMC3654430

[ref95] Vragović K. Sela A. Friedlander-Shani L. Fridman Y. Hacham Y. Holland N.. (2015). Translatome analyses capture of opposing tissue-specific brassinosteroid signals orchestrating root meristem differentiation. Proc. Natl. Acad. Sci. U. S. A. 112, 923–928. doi: 10.1073/pnas.1417947112, PMID: 25561530 PMC4311806

[ref96] Walker P. Girard I. Giesbrecht S. Whyard S. Fernando D. de Kievit T.. (2021). Tissue-specific mRNA profiling of the *Brassica napus-Sclerotinia sclerotiorum* interaction uncovers novel regulators of plant immunity. bioRxiv. doi: 10.1101/2021.03.27.437327 35961003

[ref102] Wang B. Zhu X. Wei X. (2021). Genome-wide identification, structural analysis, and expression profiles of the BZR gene family in tomato. J. Plant Biochem. Biotechnol. 1–12. doi: 10.1007/s13562-021-00711-y

[ref101] Wang D. Zhang Y. Zhang Z. Zhu J. Yu J. (2010). KaKs_Calculator 2.0: a toolkit incorporating gamma-series methods and sliding window strategies. Genomics Proteomics Bioinformatics 8, 77–80. doi: 10.1016/s1672-0229(10)60008-3, PMID: 20451164 PMC5054116

[ref99] Wang W. Sun Y.-Q. Li G.-L. Zhang S.-Y. (2019). Genome-wide identification, characterization, and expression patterns of the BZR transcription factor family in sugar beet (*Beta vulgaris* L.). BMC Plant Biol. 19:191. doi: 10.1186/s12870-019-1783-1, PMID: 31072335 PMC6506937

[ref98] Wang Y. Sun F. Cao H. Peng H. Ni Z. Sun Q.. (2012a). TamiR159 directed wheat TaGAMYB cleavage and its involvement in anther development and heat response. PLoS One 7:e48445. doi: 10.1371/journal.pone.0048445, PMID: 23133634 PMC3486836

[ref100] Wang Y. Tang H. DeBarry J. D. Tan X. Li J. Wang X.. (2012b). MCScanX: a toolkit for detection and evolutionary analysis of gene synteny and collinearity. Nucleic Acids Res. 40:e49. doi: 10.1093/nar/gkr1293, PMID: 22217600 PMC3326336

[ref97] Wang Z.-Y. Nakano T. Gendron J. He J. Chen M. Vafeados D.. (2002). Nuclear-localized BZR1 mediates brassinosteroid-induced growth and feedback suppression of brassinosteroid biosynthesis. Dev. Cell 2, 505–513. doi: 10.1016/s1534-5807(02)00153-3, PMID: 11970900

[ref103] Wei Z. Li J. (2016). Brassinosteroids regulate root growth, development, and symbiosis. Mol. Plant 9, 86–100. doi: 10.1016/j.molp.2015.12.003, PMID: 26700030

[ref105] Wu P. Song X. Wang Z. Duan W. Hu R. Wang W.. (2016a). Genome-wide analysis of the BES1 transcription factor family in Chinese cabbage (*Brassica rapa* ssp. pekinensis). Plant Growth Regul. 80, 291–301. doi: 10.1007/s10725-016-0166-y

[ref104] Wu X. Chen J. Xu S. Zhu Z. Zha D. (2016b). Exogenous 24-epibrassinolide alleviates zinc-induced toxicity in eggplant (*Solanum melongena* L.) seedlings by regulating the glutathione-ascorbate-dependent detoxification pathway. J. Hortic. Sci. Biotechnol. 91, 412–420. doi: 10.1080/14620316.2016.1162030

[ref106] Xie L. Yang C. Wang X. (2011). Brassinosteroids can regulate cellulose biosynthesis by controlling the expression of CESA genes in *Arabidopsis*. J. Exp. Bot. 62, 4495–4506. doi: 10.1093/jxb/err164, PMID: 21617247 PMC3170551

[ref107] Xu M. Ma H. Zeng L. Cheng Y. Lu G. Xu J.. (2015). The effect of waterlogging on yield and seed quality at the early flowering stage in *Brassica napus* L. Field Crop Res. 180, 238–245. doi: 10.1016/j.fcr.2015.06.007

[ref110] Yang J. Yan R. Roy A. Xu D. Poisson J. Zhang Y. (2015). The I-TASSER suite: protein structure and function prediction. Nat. Methods 12, 7–8. doi: 10.1038/nmeth.3213, PMID: 25549265 PMC4428668

[ref109] Yang X. Bai Y. Shang J. Xin R. Tang W. (2016). The antagonistic regulation of abscisic acid-inhibited root growth by brassinosteroids is partially mediated via direct suppression of ABSCISIC ACID INSENSITIVE 5 expression by BRASSINAZOLE RESISTANT 1. Plant Cell Environ. 39, 1994–2003. doi: 10.1111/pce.12763 27149247

[ref108] Yang Z. (2007). PAML 4: phylogenetic analysis by maximum likelihood. Mol. Biol. Evol. 24, 1586–1591. doi: 10.1093/molbev/msm088, PMID: 17483113

[ref111] Ye Q. Zhu W. Li L. Zhang S. Yin Y. Ma H.. (2010). Brassinosteroids control male fertility by regulating the expression of key genes involved in *Arabidopsis* anther and pollen development. Proc. Natl. Acad. Sci. U. S. A. 107, 6100–6105. doi: 10.1073/pnas.0912333107, PMID: 20231470 PMC2851861

[ref112] Yi X. Zhang Z. Ling Y. Xu W. Su Z. (2015). PNRD: a plant non-coding RNA database. Nucleic Acids Res. 43, D982–D989. doi: 10.1093/nar/gku1162, PMID: 25398903 PMC4383960

[ref113] Yin Y. Wang Z.-Y. Mora-Garcia S. Li J. Yoshida S. Asami T.. (2002). BES1 accumulates in the nucleus in response to brassinosteroids to regulate gene expression and promote stem elongation. Cell 109, 181–191. doi: 10.1016/s0092-8674(02)00721-3, PMID: 12007405

[ref114] Yu H. Feng W. Sun F. Zhang Y. Qu J. Liu B.. (2018). Cloning and characterization of BES1/BZR1 transcription factor genes in maize. Plant Growth Regul. 86, 235–249. doi: 10.1007/s10725-018-0424-2

[ref115] Yu X. Li L. Zola J. Aluru M. Ye H. Foudree A.. (2011). A brassinosteroid transcriptional network revealed by genome-wide identification of BESI target genes in *Arabidopsis thaliana*. Plant J. 65, 634–646. doi: 10.1111/j.1365-313X.2010.04449.x, PMID: 21214652

[ref117] Zhang C. Bai M.-Y. Chong K. (2014). Brassinosteroid-mediated regulation of agronomic traits in rice. Plant Cell Rep. 33, 683–696. doi: 10.1007/s00299-014-1578-7, PMID: 24667992 PMC3988522

[ref122] Zhang L. Zou J. Li S. Wang B. Raboanatahiry N. Li M. (2019). Characterization and expression profiles of miRNAs in the triploid hybrids of *Brassica napus* and *Brassica rapa*. BMC Genomics 20:649. doi: 10.1186/s12864-019-6001-x, PMID: 31412776 PMC6694508

[ref119] Zhang L. W. Song J. B. Shu X. X. Zhang Y. Yang Z. M. (2013a). miR395 is involved in detoxification of cadmium in *Brassica napus*. J. Hazard. Mater. 250, 204–211. doi: 10.1016/j.jhazmat.2013.01.053, PMID: 23454459

[ref120] Zhang X. D. Sun J. Y. You Y. Y. Song J. B. Yang Z. M. (2018). Identification of Cd-responsive RNA helicase genes and expression of a putative BnRH 24 mediated by miR158 in canola (*Brassica napus*). Ecotoxicol. Environ. Saf. 157, 159–168. doi: 10.1016/j.ecoenv.2018.03.081, PMID: 29621707

[ref116] Zhang Y. (2008). I-TASSER server for protein 3D structure prediction. BMC Bioinformatics 9:40. doi: 10.1186/1471-2105-9-40, PMID: 18215316 PMC2245901

[ref118] Zhang Y. Li B. Xu Y. Li H. Li S. Zhang D.. (2013b). The cyclophilin CYP20-2 modulates the conformation of BRASSINAZOLE-RESISTANT1, which binds the promoter of FLOWERING LOCUS D to regulate flowering in *Arabidopsis*. Plant Cell 25, 2504–2521. doi: 10.1105/tpc.113.110296, PMID: 23897924 PMC3753379

[ref121] Zhang Y. Zhang Y.-J. Yang B.-J. Yu X.-X. Wang D. Zu S.-H.. (2016). Functional characterization of GmBZL2 (AtBZR1 like gene) reveals the conserved BR signaling regulation in *Glycine max*. Sci. Rep. 6:31134. doi: 10.1038/srep31134, PMID: 27498784 PMC4976319

[ref123] Zhao Y.-T. Wang M. Fu S.-X. Yang W.-C. Qi C.-K. Wang X.-J. (2012). Small RNA profiling in two *Brassica napus* cultivars identifies microRNAs with oil production-and development-correlated expression and new small RNA classes. Plant Physiol. 158, 813–823. doi: 10.1104/pp.111.187666, PMID: 22138974 PMC3271769

[ref124] Zhiponova M. K. Vanhoutte I. Boudolf V. Betti C. Dhondt S. Coppens F.. (2013). Brassinosteroid production and signaling differentially control cell division and expansion in the leaf. New Phytol. 197, 490–502. doi: 10.1111/nph.12036, PMID: 23253334

[ref125] Zhu J.-Y. Sae-Seaw J. Wang Z.-Y. (2013). Brassinosteroid signalling. Development 140, 1615–1620. doi: 10.1242/dev.060590, PMID: 23533170 PMC3621480

