# Supplementary material for: Genome-Wide Prediction, Functional Divergence, and Characterization of Stress-Responsive BZR Transcription Factors in B. napus
Source: Front Plant Sci. 2022 Jan 4;12:790655. doi: 10.3389/fpls.2021.790655 (PMC8764130; doi:10.3389/fpls.2021.790655)
Supplement: Supplementary file 1 [file Data_Sheet_1.PDF]

Supplementary Figure 1

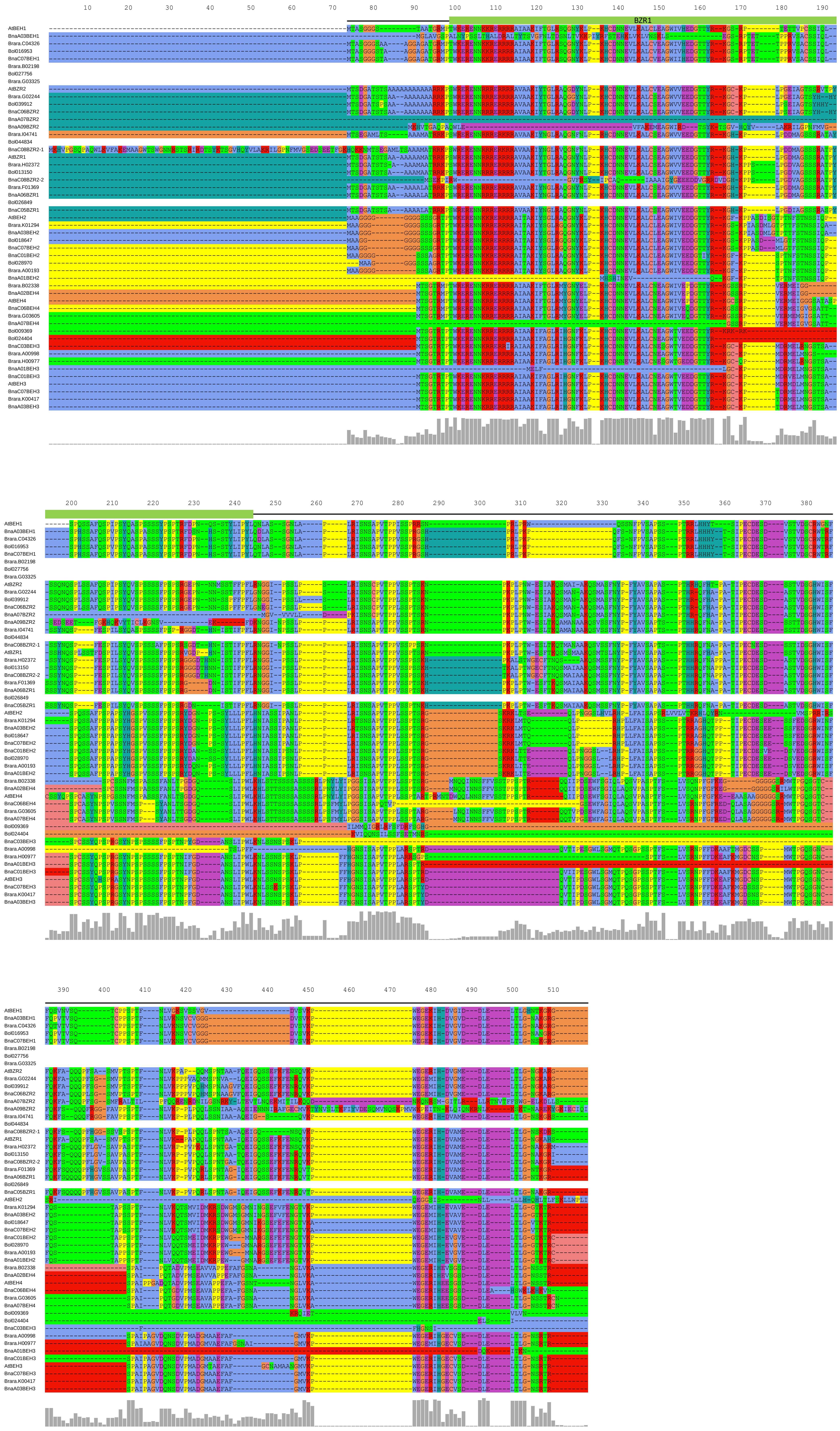

Figure S1|Multiple Sequence alignment of *A. thaliana, B. napus, B. rapa and B. oleracea BZR* gene family. The BZR1 domain is substantially conserved between four *Brassicaceae* species. Amino acid residues that are completely conserved are highlighted in different colors.
