# Supplementary figures and images for: Genome-Wide Prediction, Functional Divergence, and Characterization of Stress-Responsive BZR Transcription Factors in B. napus
Source: Front Plant Sci. 2022 Jan 4;12:790655. doi: 10.3389/fpls.2021.790655 (PMC8764130; doi:10.3389/fpls.2021.790655)

Supplementary Figure S2

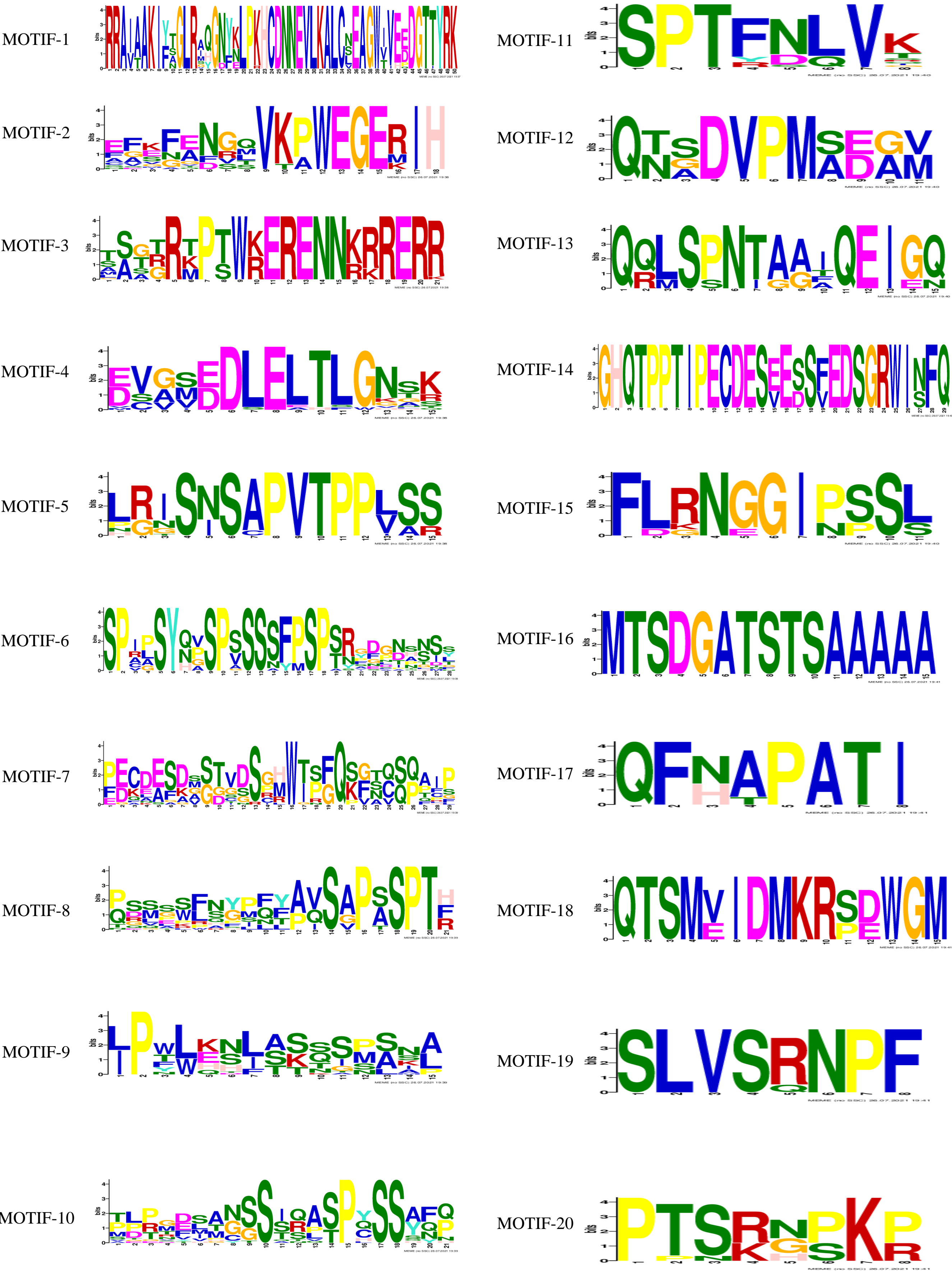

Figure S2| Schematic representation of *BnBZR*s motifs logos.

Supplement: Supplementary file 2 [file Data_Sheet_2.PDF]
