# Supplementary material for: Genome-Wide Prediction, Functional Divergence, and Characterization of Stress-Responsive BZR Transcription Factors in B. napus
Source: Front Plant Sci. 2022 Jan 4;12:790655. doi: 10.3389/fpls.2021.790655 (PMC8764130; doi:10.3389/fpls.2021.790655)
Supplement: Supplementary file 4 [file Data_Sheet_4.PDF]

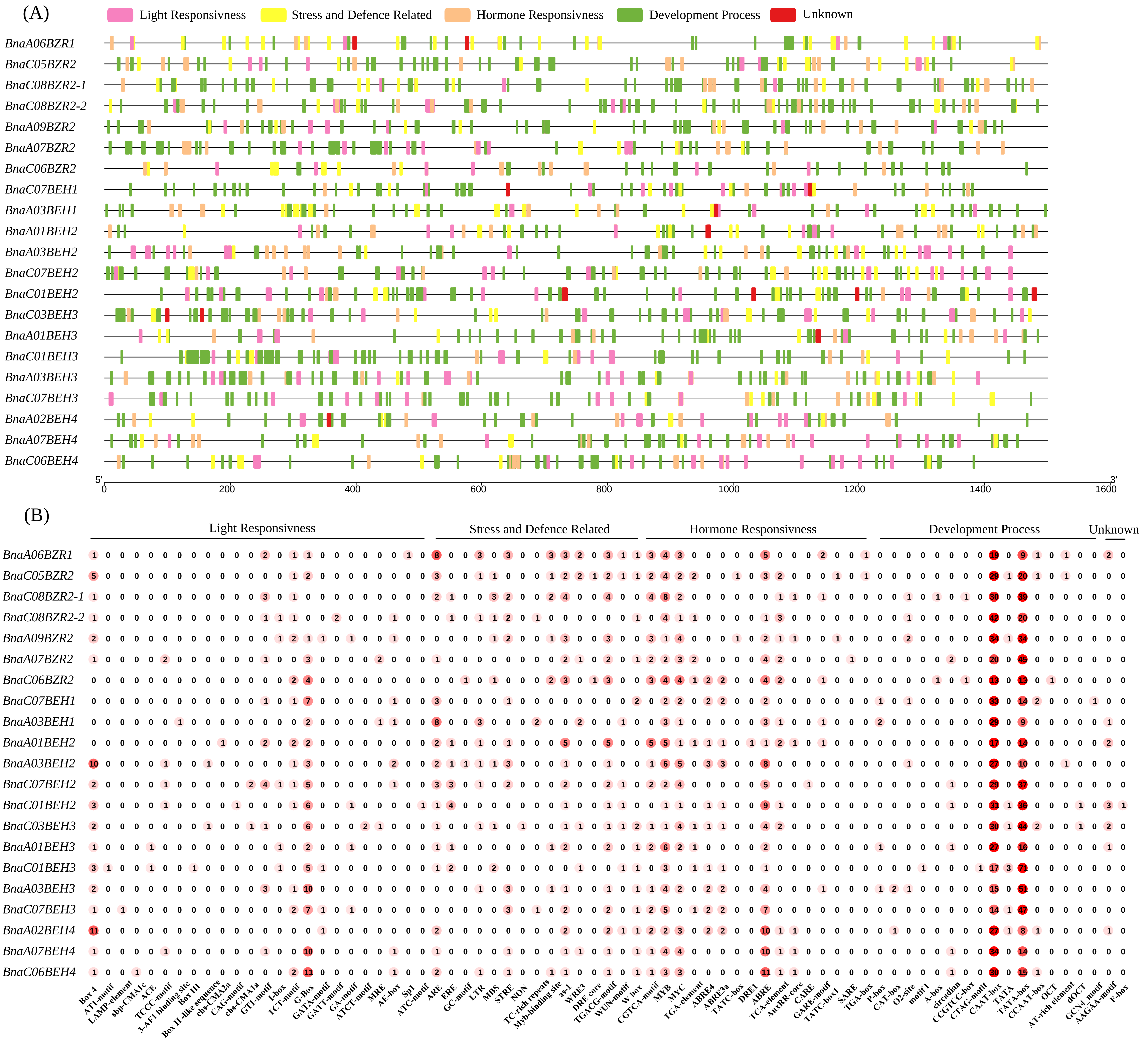

Figure S4| *cis*-element analysis and their distribution on the promoter region of the *BnaBZR* gene family. **(A)**: Distribution of cis elements in the promoter regions, different colored lines indicate the type of *cis*-element. **(B)**: Counting of *cis*-elements in each *BnaBZR* gene. Numbers in circle indicates the maximum number of *cis*-element present.
