# Supplementary material for: Genome-Wide Prediction, Functional Divergence, and Characterization of Stress-Responsive BZR Transcription Factors in B. napus
Source: Front Plant Sci. 2022 Jan 4;12:790655. doi: 10.3389/fpls.2021.790655 (PMC8764130; doi:10.3389/fpls.2021.790655)
Supplement: Supplementary file 5 [file Data_Sheet_5.PDF]

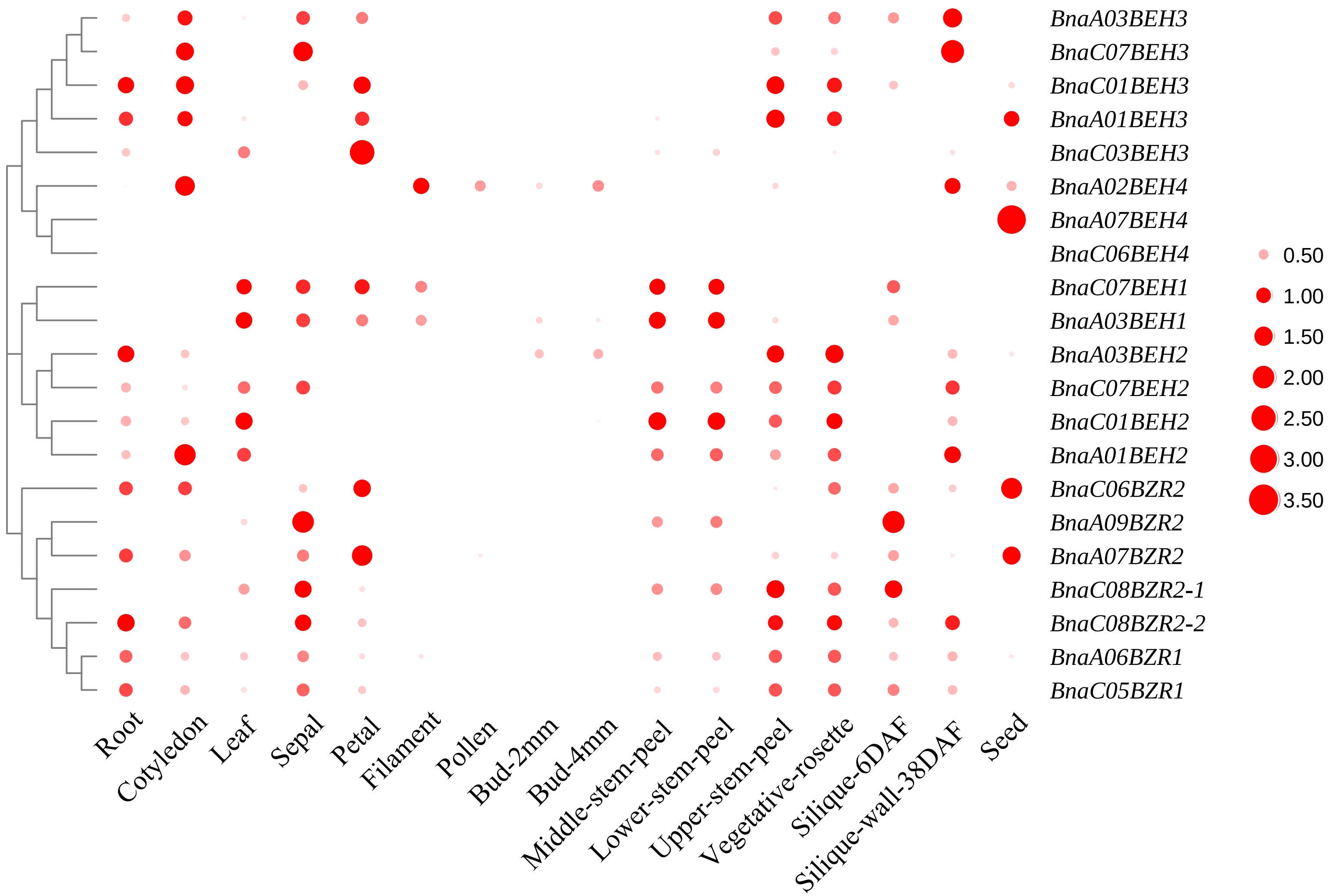


**Figure S5|** Heatmap of the expression profile of *BnaBZRs* in different tissues**.** The color scale reflects the data of the expression being processed with normalization of log2 listed in Table S8.1, Different diameters of the circle represent the higher and lower expression.
