# Supplementary material for: Genome-Wide Prediction, Functional Divergence, and Characterization of Stress-Responsive BZR Transcription Factors in B. napus
Source: Front Plant Sci. 2022 Jan 4;12:790655. doi: 10.3389/fpls.2021.790655 (PMC8764130; doi:10.3389/fpls.2021.790655)
Supplement: Supplementary file 6 [file Data_Sheet_6.PDF]

Supplementary Figure S4

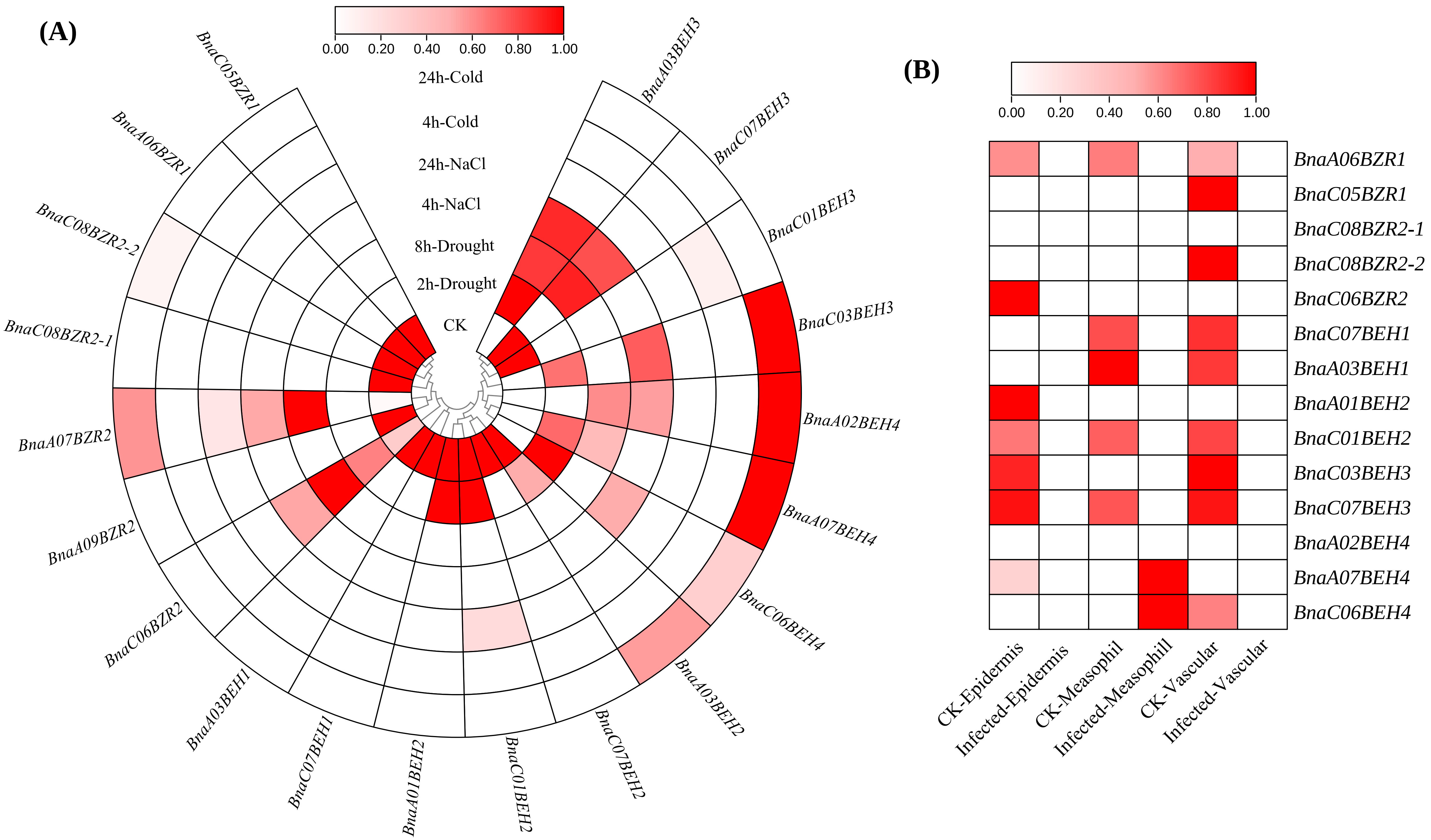

Figure S6| Transcriptomic study of the *BnaBZRs* expression under different stresses. Log2 normalization was used to evaluate the expression data. The color scale bar represents relative expression levels ranging from high to lower expression (Table S9.1).
