# Supplementary material for: Genome-Wide Prediction, Functional Divergence, and Characterization of Stress-Responsive BZR Transcription Factors in B. napus
Source: Front Plant Sci. 2022 Jan 4;12:790655. doi: 10.3389/fpls.2021.790655 (PMC8764130; doi:10.3389/fpls.2021.790655)
Supplement: Supplementary file 7 [file Data_Sheet_7.PDF]

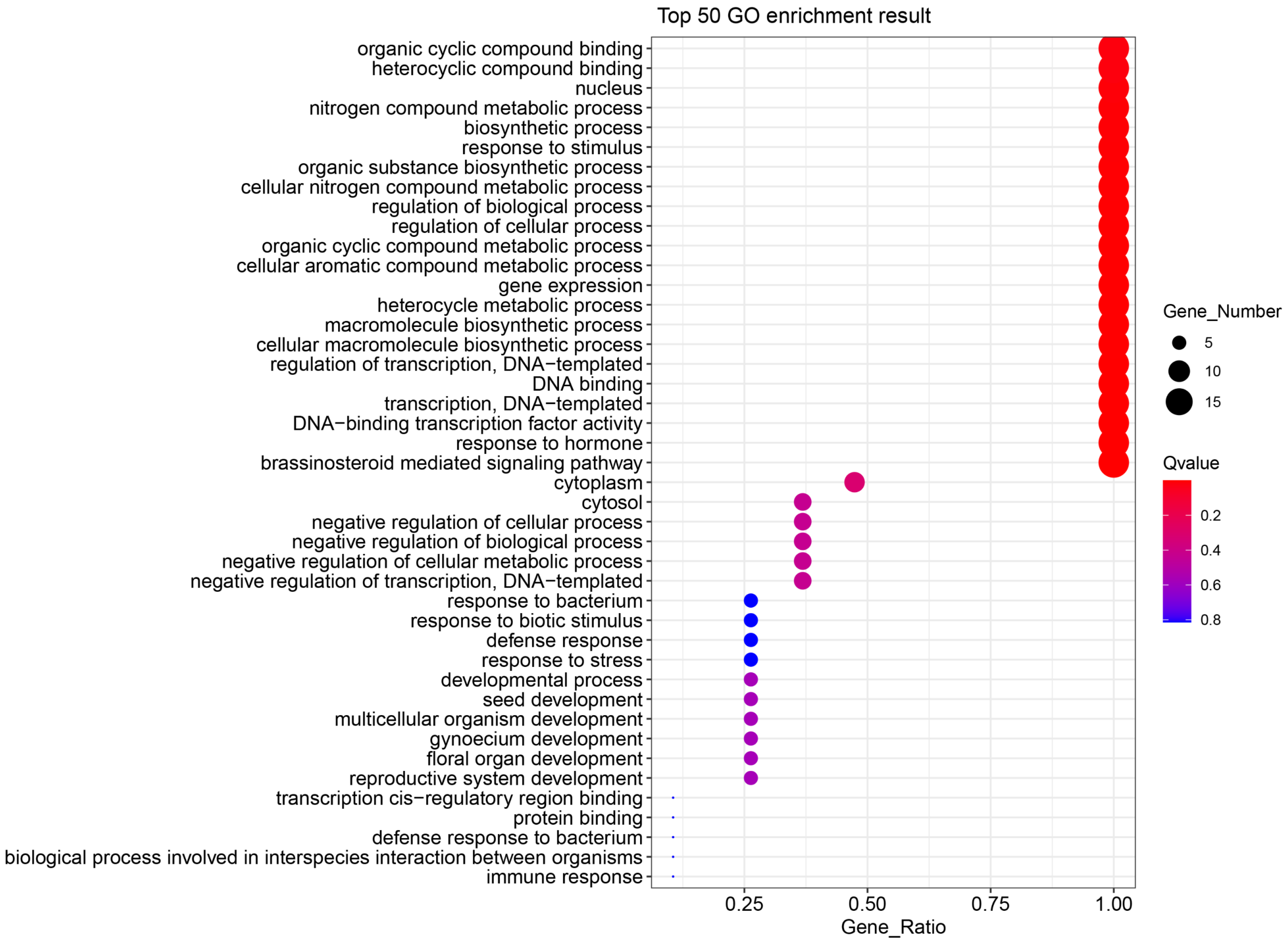

Figure S7| Gene Ontology **(**GO) enrichment analysis of the *BZR* gene family. X-axis indicates the entry name. Y-axis indicates the gene ratio. Dot size represents the number of genes enriched in the entry, and the different color of dot represents the q-score.
